# Ligand-Independent Traffic of Notch Buffers Activated Armadillo in *Drosophila*

**DOI:** 10.1371/journal.pbio.1000169

**Published:** 2009-08-11

**Authors:** Phil G. T. Sanders, Silvia Muñoz-Descalzo, Tina Balayo, Frederik Wirtz-Peitz, Penelope Hayward, Alfonso Martinez Arias

**Affiliations:** Department of Genetics, University of Cambridge, Cambridge, United Kingdom; Harvard Medical School, Howard Hughes Medical Institute, United States of America

## Abstract

Notch receptors act as ligand-dependent membrane-tethered transcription factors with a prominent role in binary cell fate decisions during development, which is conserved across species. In addition there is increasing evidence for other functions of Notch, particularly in connection with Wnt signalling: Notch is able to modulate the activity of Armadillo/ß-catenin, the effector of Wnt signalling, in a manner that is independent of its transcriptional activity. Here we explore the mechanism of this interaction in the epithelium of the *Drosophila* imaginal discs and find that it is mediated by the ligand-independent endocytosis and traffic of the Notch receptor. Our results show that Notch associates with Armadillo near the adherens junctions and that it is rapidly endocytosed promoting the traffic of an activated form of Armadillo into endosomal compartments, where it may be degraded. As Notch has the ability to interact with and downregulate activated forms of Armadillo, it is possible that in vivo Notch regulates the transcriptionally competent pool of Armadillo. These interactions reveal a previously unknown activity of Notch, which serves to buffer the function of activated Armadillo and might underlie some of its transcription-independent effects.

## Abstract 2

Full-length Notch receptor binds to the Wnt pathway effector β-catenin and mediates its endocytosis and degradation, demonstrating a novel mechanism by which Notch may modulate Wnt pathway activity.

## Author Summary

Establishment of the correct shape and pattern of tissues within an organism requires the integration of molecular information present in signalling and transcriptional networks and demands delicate exchanges and balances of their activities. A large body of experimental work has revealed close correlations in the activities of two pathways: Notch and Wnt, which suggest the existence of multiple links between them. Notch signalling relies in part upon the activity of the Notch protein, a membrane-bound receptor with a transcription factor domain that can be released from the membrane by proteolytic cleavage. On the other hand Wnt proteins are ligands that trigger changes in the activity of ß-catenin, which is called Armadillo in the fruit fly *Drosophila melanogaster*. In this study we uncover a previously unknown activity for Notch: endocytosis and trafficking of full length Notch, which targets Armadillo for degradation. This activity of Notch is independent of its ligands, Delta and Serrate, and of its downstream effector, the transcription factor Suppressor of Hairless. We further show that in the absence of Notch, which has been shown to act as a tumor suppressor in mammals, expression of an activated form of Armadillo causes tissue overgrowth and changes in the polarity of cells. Our results suggest that *Drosophila* Notch can promote the degradation of activated forms of Armadillo and may buffer cells against fluctuations in Wnt signalling activity.

## Introduction

The *Notch* gene of *Drosophila* encodes a member of a family of conserved single transmembrane receptors with key tasks in the information processing activity of animal cells [1]–[4]. They are involved in a wide variety of processes during development but their best characterized function is in the process of lateral inhibition and related events, in which Notch signalling is used to choose between two alternative cell fates in a context dependent manner [4]–[6]. There are two prominent structural features that define the family: a tandem array of EGF repeats in the extracellular domain that act as docking sites for ligands to trigger and modulate the activity of Notch, and seven ankyrin (ANK) repeats in the intracellular domain that provide a major face for interactions with effectors [7]–[14].

It is well established that Notch acts as membrane-tethered transcription factor (reviewed in [1],[4],[15]). Binding of members of the DSL (Delta, Serrate, Lag1) family of Notch ligands to specific EGF-like repeats leads to the shedding of most of the extracellular domain and triggers a sequence of proteolytic cleavages in the membrane proximal region, which result in the release of the Notch intracellular domain (Nintra) from the membrane [1],[15]–[19]. Nintra accesses the nucleus where it modulates transcription through interactions with a member of the CSL (CBF in mammals, Su(H) in *Drosophila*, and Lag2 in *C. elegans*) family of transcription factors, and Mastermind (MAML in vertebrates) [20]–[22]. The interactions between Notch and CSL are mediated by the ANK repeats [11],[23] and result in the activation of specific target genes. Recently, a number of studies, particularly in *Drosophila*, suggest that endocytosis and traffic of Notch are required for the generation and activity of Nintra [24]–[33].

Inappropriate activation of Notch signalling has been associated with a number of tumours in humans; in particular with T-cell acute lymphoblastic/lymphoma (ALL) leukemias, where activating mutations in *Notch* have been found to be linked to the disease [34]–[36]. However, there is also evidence that Notch can act as a tumour suppressor [37]–[40]. In one instance, this tumour suppressor function has been associated with signalling by ß-catenin, the effector of Wnt signalling [38],[39]. Functional interactions between Wnt and Notch signalling have been reported frequently (reviewed in [3]) and are underpinned by biochemical studies that identify Dishevelled, GSK3ß, and ß-catenin, all key elements of the canonical Wnt signalling pathway, as Notch interacting proteins [3],[41]–[47]. Although in many instances these interactions probably reflect the convergence of the two signalling pathways onto common target genes, studies in *Drosophila* have shown that Notch can modulate Wnt signalling in an Su(H)-independent manner by targeting Armadillo, the *Drosophila* homologue of ß-catenin [43],[45],[48]–[50].

Here we explore the mechanism of the interaction between Notch and Wnt signalling in *Drosophila*. We find that in the absence of *Notch*, an activated form of Armadillo promotes changes in the proliferative and adhesive properties of epithelial cells in *Drosophila*. This observation reveals an effect of Notch on Wnt signalling that is independent of its ligands and the activity of Su(H), but requires the endocytosis and traffic of Notch. Our results provide a mechanism for the interaction between Notch and Wnt signalling that has implications for the homeostasis of the cell and, perhaps for the development of tumours.

## Results

### Notch Modulates Cell Proliferation, Growth, and Polarity in the Imaginal Disc Epithelia of *Drosophila*

Experiments in *Drosophila* have suggested that Notch can modulate the activity of Armadillo in an Su(H)-independent manner [43],[48]–[50]. This observation is reminiscent of the situation in the skin of the mouse where loss of *Notch1* function leads to elevated levels of ß-catenin and sensitizes the tissue to the development of basal cell carcinomas [38],[39]. For this reason and to explore further the mechanism of the interaction between Notch and Armadillo, we expressed in the imaginal discs an activated form of Armadillo, Arm^S10^ (a GSK3ß insensitive form of Armadillo that promotes constitutive Wnt signalling [51] in cells mutant for *Notch*).

**Figure 1:**
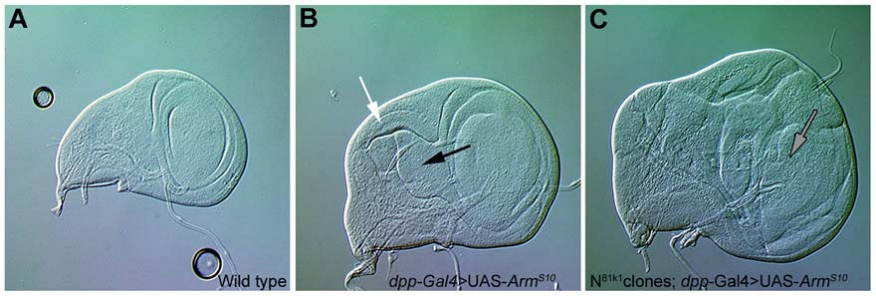
Armadillo induces outgrowths in the absence of Notch. Nomarski images of third instar wing discs. (A) Wild-type wing imaginal disc. (B) Wing disc expressing UAS*-Arm^S10^* under the control of *dpp*-Gal4. Notice the expansion of the hinge region (black arrow) and the associated deformation of the wing pouch. There is also a slight overgrowth in the scutellar region (white arrow). (C) Wing disc heterozygous for *Notch*, with FRT/FLP induced clones of *Notch* mutant cells expressing UAS*-Arm^S10^* under the control of *dpp*-Gal4. Notice the outgrowths with tumorous appearance (grey arrows) masking the normal features of the wing disc. All pictures taken at the same magnification.

Loss of Notch function during the development of the wing results in stage-dependent altered growth rates and patterning defects, with little evidence of an increased activity of Armadillo (Figures 1A and S1A) [43],[52],[53]. This observation could be due to the loss of the Su(H)-dependent activity, which might mask additional consequences of the loss of function of Notch in this system. In contrast to the effects of loss of Notch function, gain or loss of Wnt signalling has only subtle effects on the growth of the wing primordium [54]–[57], and expression of Arm^S10^ (along the anterior-posterior [AP] boundary using *dpp*-Gal4 driver) results in changes in gross morphology and alterations in cell fate in a region-specific manner with little or no effect on the overall size of the wing pouch or cell proliferation (Figures 1B and S1B) [43],[53],[55],[57]–[59]. However in the absence of Notch, expression of Arm^S10^ produces outgrowths in the wing discs (Figures 1C and S1C), which are reminiscent of the effects of mutations in *lgd*, *exp*, and *mer*, which have been linked with tumour suppression in *Drosophila* [26],[60]. In addition, there are some effects on cell fate, e.g., in the notum neural development is observed in regions outside the proneural clusters where Armadillo gain of function or Notch loss of function on their own have little or no effect (Figure S1D–S1F). In these experiments the clones are generated continuously, using the FRT/FLP system, and therefore the effect is a cumulative average of clones generated at different times and different places.

**Figure 2:**
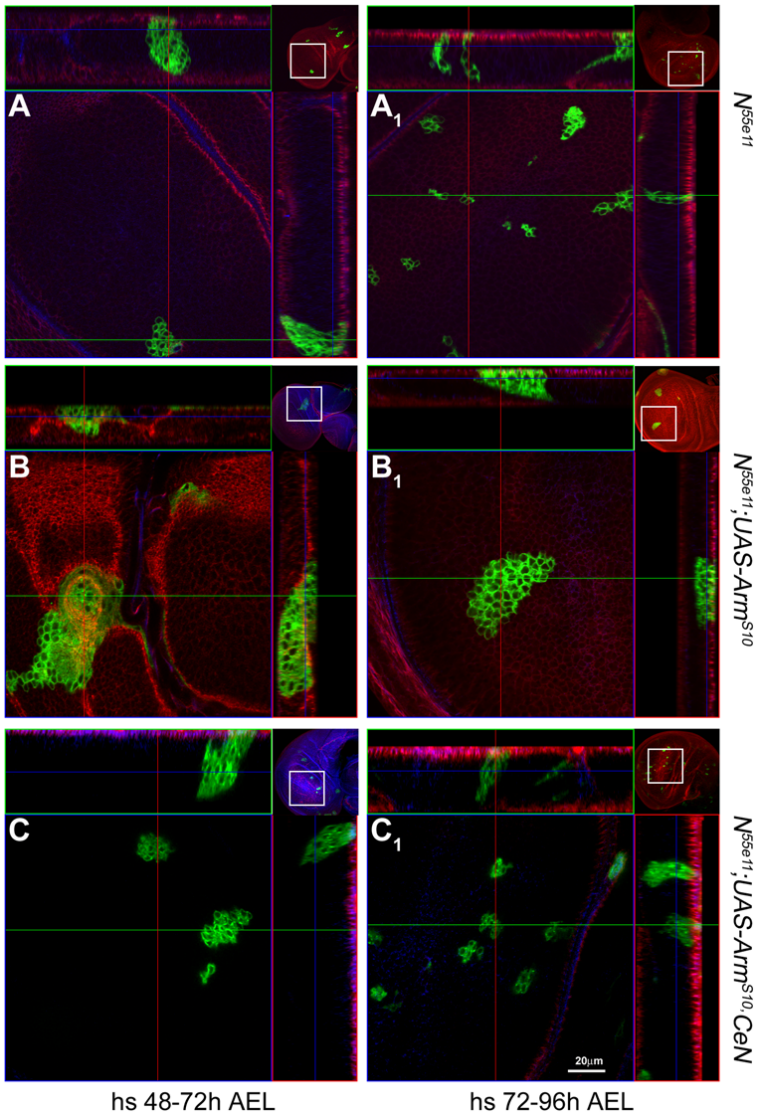
Armadillo induces defects in cell proliferation and adhesion in the absence of Notch. Confocal images of third instar wings discs with MARCM clones (labelled in green) of *Notch* mutant cells (A and A_1_), *Notch* mutant cells that overexpress Arm^S10^ (B and B_1_), and *Notch* mutant cells that overexpress Arm^S10^ and CeN, induced at 48–72 h (A–C) or 72–96 h AEL (A_1_–C_1_). The small insets at the corners of each image are low magnification pictures of the discs shown which act as a reference (see Figure S4 for larger images of these insets). In this and the following images of related experiments, the pictures on the top and the right represent optical z-sections through the clones following the green and the red lines shown in the main picture. Notice that the clones expressing Arm^S10^ are much larger and also show a rounded appearance with little or no contacts with the apical and basal regions of the epithelium. This is particularly clear in clones induced in the 48–72 h period. In some instances one can observe large clones in the peripodial membrane, which exhibit the unusual feature of fusing with clones generated in the disc epithelium. See Figures S2 and S3, and Videos S1 and S2, for more details of the outgrowths. The expression of CeN in the clones of *Notch* mutant cells that overexpress Arm^S10^ reduces the proliferation effect, and corrects the loss of basal connection and the adhesion defects. See Video S3 for the complete z-stack on the *N^55e11^*;UAS*-Arm^S10^,CeN *clone shown in (C). The red channel shows Scribble (a basolateral cell junction marker) and the blue, the DCadherin staining (an adherens junction marker). All pictures taken at the same magnification. Scale bar in (C_1_), 20 µm.

In order to explore the origin and fate of these outgrowths in more detail, we overexpressed Arm^S10^ in clones of *Notch* mutant cells generated at defined times in development using the MARCM method [61]. Clones of *Notch* mutant cells generated early in larval development are never recovered, probably because of competition by surrounding wild-type cells, and with later inductions the number and size of clones of *Notch* mutant cells observed increases, though it never reaches the figures of wild-type clones [52] (Figures 2A, S1A, S4A, and S7C for *Notch* clones and S5A for wild-type clones). In general the clones of Notch mutant cells are not frequent and do not grow well. Expression of Arm^S10^ in *Notch* mutant cells rescues the viability of the early generated clones (24–48 h after egg laying [AEL], Figure S2 and Video S1) and leads to tightly packed spheres with large numbers of cells and abnormal polarity. There is usually a single large sphere per disc, which tends to be positioned on the edges of the disc suggesting a tendency of the cells to sort from the surrounding ones. Clones of cells mutant for *Notch* expressing Arm^S10^ induced between 48–72 h also give rise to sphere-like structures with large numbers of cells similar to the early ones (Figures 2B and S3B; Video S2), but those induced after 72 h appear scattered through the tissue, lose basal contact, and exhibit a variety of organizations (Figure 2B_1_, and for details and discussion, Figures S2 and S3). It is reasonable to think that the later-induced clones represent the early events in the process of formation of the spheres of cells, and this suggests that coalescence of different clones is a component of the phenotype. These overgrown aggregates are not restricted to the wing pouch as they can also be found in the notum as well as in other discs and, interestingly, in the peripodial membrane where cells lose their characteristic flat epithelial appearance and can fuse with the cells of the wing epithelium (Figure S3B and unpublished data).

Altogether, these observations suggest that loss of function of *Notch* unlocks a potential for Arm^S10^ to regulate cell proliferation, polarity, and adhesion. Some of this activity might be mediated by canonical Wnt signalling, and the clones of Notch display elevated levels of Arm^S10^ and, most significantly, elevated levels in the nuclei (Figure S6).

### Loss of Delta, Serrate, or Su(H) Exerts Different Effects on the Activity of Armadillo

There are suggestions that the activity of Notch that regulates Wnt signalling does not require the biochemical events associated with ligand-dependent cleavage and transcriptional activity of Notch [43],[45],[49],[50]. To test this further we assessed the effects of expressing Arm^S10^ in clones of cells lacking the Notch ligands, Delta and Serrate, as well as its transcriptional effector Su(H).

**Figure 3:**
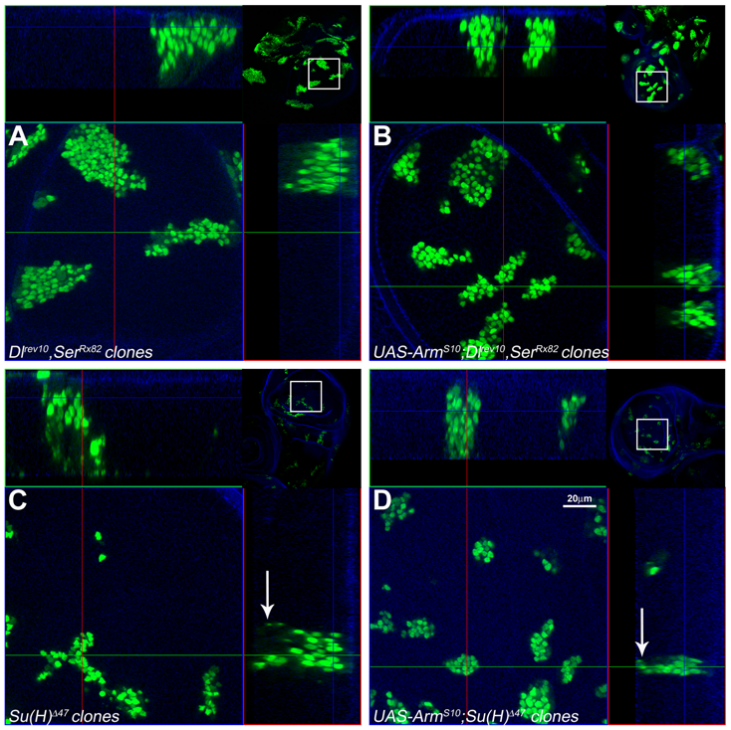
Absence of the Notch ligands Delta and Serrate or the transcriptional effector Su(H) does not affect the activity of Armadillo. Confocal images of third instar wings discs with clones of cells induced at 48–72-h AEL mutant for both *Dl^rev10^* and *Ser^Rx82^* (A and B) or *Su(H)* ^Δ*47*^ (C and D), without and with Arm^S10^ as indicated. Clones of cells mutant for *Dl^rev10^* and *Ser^Rx82^* (A) grow and remain integrated in the epithelium maintaining their apicobasal polarity as can be seen in the optical z-section. No major changes are observed when Arm^S10^ is expressed in these cells (B). Clones of cells mutant for *Su(H)* ^Δ*47*^ display a number of distinctive features ([C] and see Video S4). They are smaller and appear more dispersed than those of cells mutant for *Dl^rev10^* and *Ser^Rx82^*. The edges of the clones are ragged and irregular and give the impression that the cells are dispersing. Most notably cells can be observed dispersed within the plane of the epithelium with a large amount of apoptotic cells on the basal side (white arrow, see optical z-section). Expression of Arm^S10^ in these cells increases the size of the clones, alters their appearance, and reduces, but does not eliminate, the number of apoptotic cells in the basal region (see Video S5). Technical details of the images as in Figure 2 with the small insets at the corners of each image being low magnification pictures of the discs shown which act as a reference. Scale bar in (D), 20 µm.

In order to study the effect of ligand-dependent signalling on the activity of Arm, we chose to express Arm^S10^ in cells that simultaneously lack Delta (Dl) and Serrate (Ser). Clones of cells doubly mutant for *Dl* and *Ser* are more frequent than *Notch* mutant clones at any stage of development, and do not exhibit obvious phenotypic alterations (Figure 3A). The differences between the two mutant conditions are further emphasized by their differential behaviour in the presence of Arm^S10^: in contrast to *Notch* mutant cells, *Dl/Ser* double mutant cells expressing Arm^S10^ remain integrated in the epithelium and do not exhibit growth defects relative to the *Dl/Ser* double mutants alone (Figure 3B). Surprisingly, clones of cells lacking *Su(H)* exhibit phenotypes that are different from both *Notch* and *Dl/Ser* double mutants: many small clones scattered over the disc, with very rugged edges and associated with a large number of dead cells in the basal side (Figure 3C and Video S4). Expression of Arm^S10^ in these clones increases their size, reduces the number of apoptotic cells, and makes the clones more rounded in appearance but cells do not lose their polarity (Figure 3D and Video S5; for details and comparisons see Figure S7). One possible interpretation for these changes is that the expression of Arm^S10^ is able to rescue some of the apoptosis caused by the loss of *Su(H)* and give rise to bigger and more organized clones.

These results are surprising as the different phenotypes caused by the loss of function of *Dl*, *Ser*, *Notch*, and *Su(H)* challenge the simple linear interaction between Notch, its ligands, and its effector. These differences and the singular phenotypes of Notch in the presence of Arm ^S10^ also emphasize that the effects of Notch on the activity of Arm are unlikely to be mediated by its Su(H)-dependent transcriptional activity.

### A Ligand-Independent Activity of Notch

**Figure 4:**
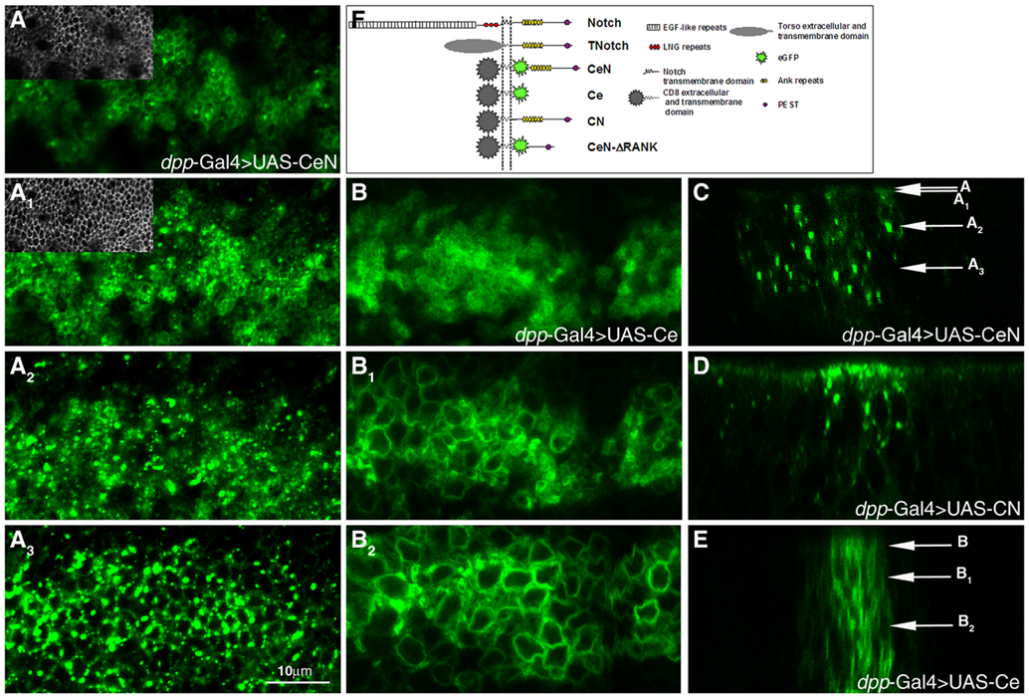
Localization of CeN, CN, and Ce in the epithelial cells of third instar wing discs. (A–A_3_) Localization of CeN at and above the adherens junctions (insets in A and A_1_ show the adherens junctions as labelled by DCad staining in the same image) as well as in more basal dots, which represent vesicles (A_2_–A_3_). The localization is revealed by the fluorescence of the eGFP. Notice that there is a pool of Notch apical to the adherens junctions. (B–B_2_) Localization of Ce in all membranes at all levels of the cell (B, apical' B_1_, subapical; B_2_, basal). This molecule contains the eGFP fused to the transmembrane and extracellular domains of CD8 and indicates that the localization of CeN is determined by the sequences of the Notch protein that are added to Ce. (C–E) Confocal optical z-sections through the wing pouch of a disc expressing CeN, showing the large vesicles of stain (C); CN, a chimera like CeN but without the eGFP that can only be visualized with an antibody against the intracellular domain of Notch, NICD, (D); and Ce, highlighting the overall and nonspecific distribution of eGFP to all membranes of the cell (E). Notice that in (D) it is not easy to distinguish the endogenous Notch from CN other than by the amount. The arrows in (C) and (E) point to the approximate levels of the pictures (A–A_3_) and (B–B_2_). The expression of the different proteins is directed by *dpp*-Gal4 in all cases. Scale bar, 10 µm. (F) Schematic of the structure of the Notch receptor and related chimeras used in this work.

The effects of loss of Notch function on the activity of Armadillo provide a clear cut experimental test for the possibility that Notch encodes more than the Su(H)-mediated activity. To do this, we tested the ability of different forms of the Notch receptor to complement the effects of loss of *Notch* function on Armadillo activity in the wing disc: a full length Notch molecule (FLN), and two membrane-tethered versions of Nintra, a CD8eGFPNotch (CeN) and CD8Notch (CN), with the extracellular and TM domain of CD8, which cannot be cleaved as they lack the intramembrane cleavage sites, and do not act through Su(H) (see Figure 4F) [48],[49].

To gain a better understanding of the activity of these molecules, we first analyzed their subcellular localization in wing imaginal discs (Figure 4). The CeN molecule, assessed using the fluorescence of enhanced green fluorescent protein (eGFP) (or antibodies against CD8, see below), localizes to a domain both apical to and overlapping with the adherens junctions, and to large intracellular puncta located throughout the cell (Figure 4A and 4C). The CN molecule can only be visualized by immunostaining, but displays a similar distribution to CeN, ruling out an effect of eGFP on the distribution and activity of the molecules (Figure 4D and unpublished data). These localizations show an overlap with that of endogenous Notch and are determined by the intracellular domain of Notch, as a control of CD8 fused to eGFP (Ce) is distributed to all membranes of the cells indiscriminately (Figure 4B). We concentrated the rest of the studies on CeN, testing for ligand- and Su(H)-independent activities of Notch and in particular for its ability to regulate the Armadillo activity in the absence of Notch, though CN has similar activities in vivo.

**Figure 5:**
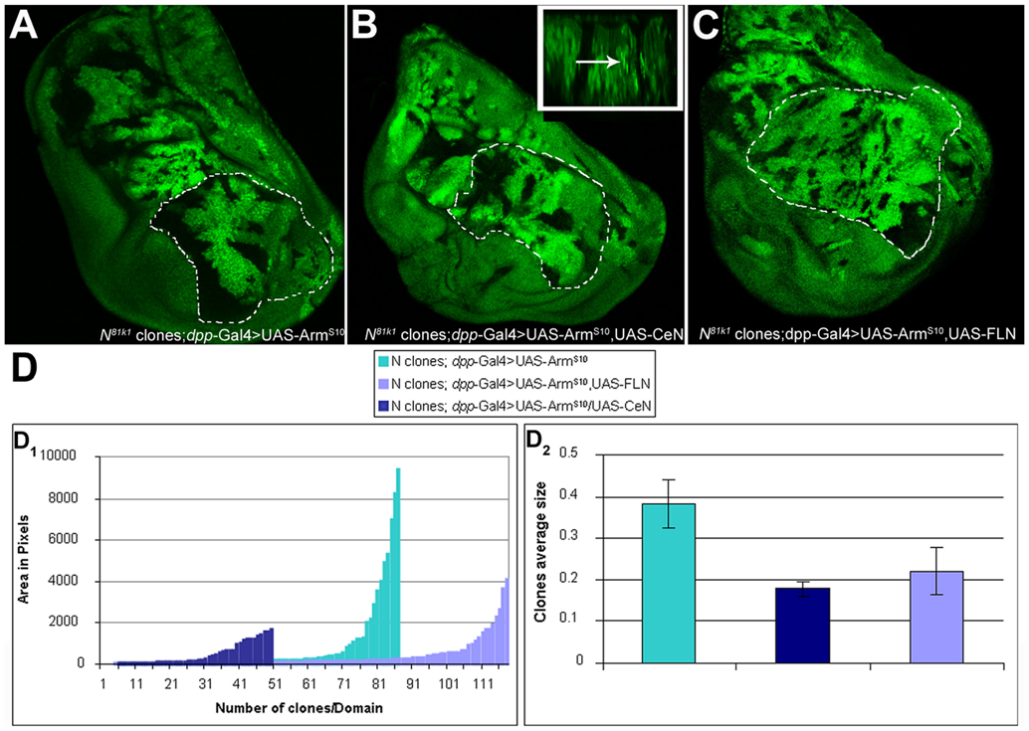
Complementation of the buffering activity of Notch. (A–C) Third instar imaginal wing discs containing *Notch* mutant clones (generated using the FRT/FLP system, labelled by the absence of GFP) and expressing UAS*-Arm^S10^* alone (A) or in combination with UAS*-CeN *(B) or UAS*-FLN *(Full-Length-Notch) (C) under the control of *dpp*-Gal4 (see Materials and Methods for details). Notice that the clones are smaller and more frequent in (B) and (C) than those in (A). This mirrors the effects of the MARCM clones but because in these experiments the clones are induced continuously there are more clones that are averaged over the whole of imaginal development. The wing pouch of (C) tends to show a bigger size, which is due to the effect of the Su(H)-dependent activity of Notch, which is provided by FLN, and which cannot be provided by CeN [58]. The dotted lines indicate the domain of the wing pouch analyzed in (D). The inset in (B) shows an optical z-section of the disc; the arrow points to CeN dots. (D) Quantification of the area of the clones in the wing pouch in the different genetic backgrounds (for details see Materials and Methods). At least three discs were analyzed per genotype. (D_1_) Distribution of the area and number of individual clones per domain. Every bar represents an individual clone with the size, expressed in pixels, indicated in the *y*-axis. The difference in numbers of clones is related to the number of experimental discs included in the count. Note that CeN and FLN reduce the area (size) of the clones. (D_2_) Relative average size of the clones analyzed in (D_1_).

Expression of CeN in cells of the wing imaginal discs results in small clones with rounded edges (Figure S5C), suggesting that it has an ability to reduce growth. The resulting adults exhibit gain of function phenotypes: loss of PNS precursors and veins (unpublished data). We also find that CeN can provide the growth suppressor activity of Notch on the activity of Arm^S10^. Clones of *Notch* mutant cells that express Arm^S10^ together with CeN are smaller than those that express Arm^S10^ alone (Figures 2C, 2C_1_, 5B, and 5D), and cells recover their polarity and adhesive properties, spreading over the disc (see also Figure S3C and S3D). To eliminate the possibility that the effects are due to a “neomorphic” activity of the CeN molecule, we repeated the experiment using FLN and observed a similar reduction in the size of the clones as observed with CeN (Figure 5C and 5D). We complemented these experiments by expressing Nintra, a form of Notch that promotes mainly the transcriptional activity, in cells that lack Notch and express Arm^S10^. The result is a combination of two phenotypes: a larger disc and, additionally, a suppression of the activity of Arm^S10^ in the clones (Figure S8). While this could be construed to suggest that the effects of Notch on Arm are mediated by Nintra, this interpretation should be considered carefully. In cultured *Drosophila* cells, Nintra can reduce the activity of Armadillo on a Wnt reporter in a manner that is independent of Su(H) [49], and this is likely to be also the case here. It is well established that in the wing, Arm and Nintra synergize [53],[58], and while this interaction can explain the large size of the discs observed in this experiment, it cannot explain the suppression of the activity of Arm^S10^ in the clones, which are now reduced in size. We surmise that this suppression is mediated by the excess of Nintra binding to proteins that interact with it, particularly Arm, and thereby neutralizes their activity.

Altogether these results argue that the tumour suppressor activity of *Notch* is an intrinsic function of Notch itself, very likely mediated by the full length receptor, and that it does not require an interaction with its ligand nor its transcriptional function.

### Ligand-Independent Traffic of Notch Modulates the Activity of Armadillo

**Figure 6:**
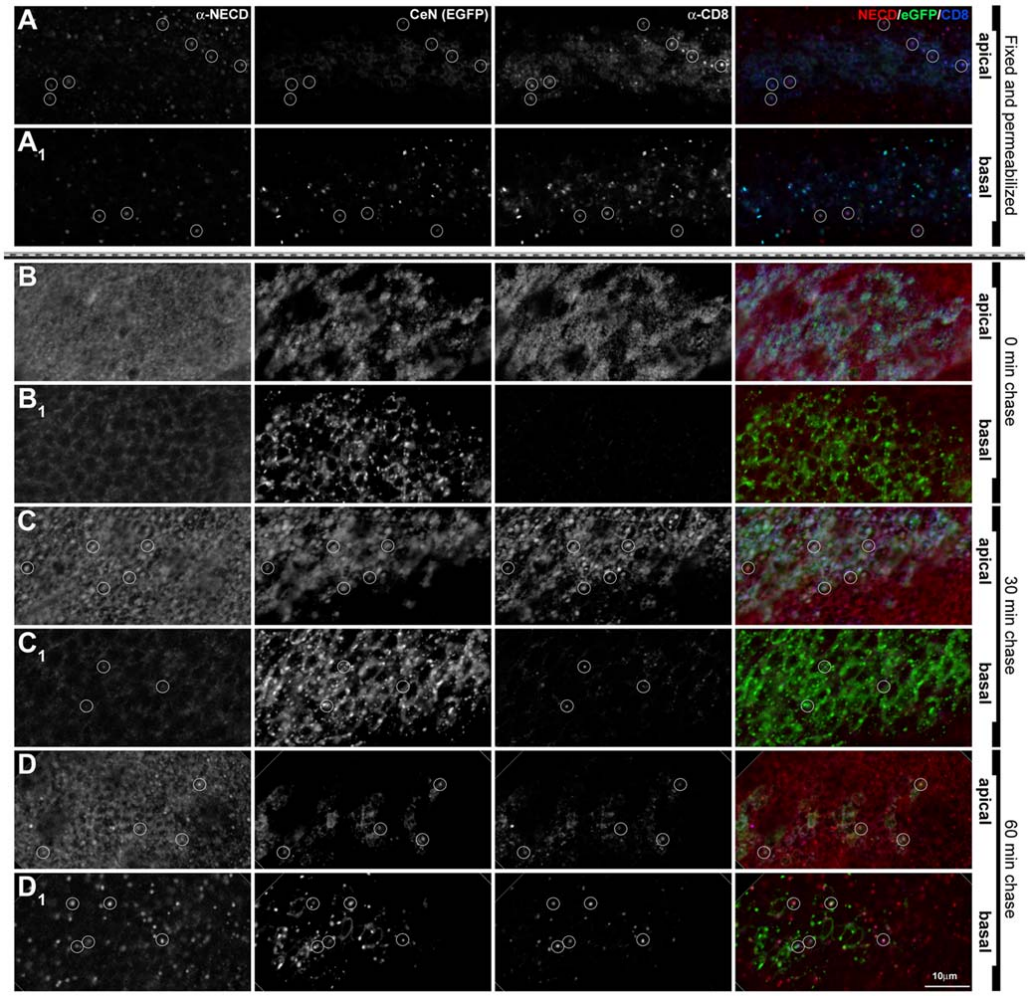
Endocytosis and traffic of CeN reflects Notch. Expression of endogenous Notch in a wing disc in which UAS*-CeN *is expressed under the control of *dpp*-Gal4. (A) Discs were fixed and permeabilized before staining with an antibody against the extracellular domain of Notch (red channel) and CD8 (blue channel). Optical sections through the apical (A) and the basal (A_1_) side of the cell. In apical levels, eGFP (green channel) and CD8 localize mostly in the membranes at the level of the adherens junctions (see also Figure 4A). Note that both in apical and basal levels the CD8 and eGFP vesicles colocalize. (B–D) Notch and CD8 tracked over time, by pulsing CeN expressing live wing discs with an antibody against the extracellular domain of Notch (red channel) and CD8 (blue channel), and chasing for 0 (B), 30 (C), and 60 min (D). (B–D, apical; B_1_–D_1_, basal sections). After 0 min of chasing, the endogenous Notch and CD8 localize in the apical membrane of the cells (B), and there are no vesicles in basal levels (B_1_). After 30 min of chase, the endogenous Notch has been cleared completely from the apical membranes and localizes in vesicles mostly in apical levels; at this time point, CD8 also goes to vesicles in the apical level, although some remain in the membrane (C–C_1_). After 60 min of chase, the endogenous Notch localizes in vesicles in apical and basal levels; at this time point, CD8 also goes to vesicles in both levels (D–D_1_). Circles mark some of the NECD, CeN, and CD8 colocalizing vesicles. In all cases the apical and basal images were taken in equivalent levels in the dorsal region of the wing pouch. The apical sections were taken at the level of the adherens junctions and the basal 7 µm underneath. See Figure S10 for an extended version of this figure. Scale bar, 10 µm.

The pattern and distribution of the CeN protein in the epithelial cells suggests that it is actively trafficking, as it is localized to the apical membrane of cells and to more basal puncta, reminiscent of vesicles, in a pattern that overlaps with that of the endogenous Notch (Figures 4A and 6A). It has been shown before that Notch can be found in endosomes [26],[33]. Therefore, for a preliminary characterization of these puncta, we investigated, whether they colocalized with several endosomal markers. We have detected a small degree of colocalization of Notch with either Rab5 or Rab7 and some more substantial localization with Rab11 (early, late, and recycling endosomal markers, respectively; Figure S9A and unpublished data). However, many of the Notch puncta colocalized with SARA (Figure S9C), an endosomal protein identified as an element of transforming growth factor-β (TGF-β) signal transduction pathway, which has been shown to regulate Notch signalling during asymmetric cell divisions [62], and with Spinster and Carnation, proteins characteristic of late endosomes (Figure S9B and unpublished data) [63]–[66]. We have confirmed that CeN trafficks by checking that it colocalizes with endocytosed Dextran (unpublished data) and, most significantly, by uptake experiments using anti-CD8 and anti-Notch antibodies to label and chase cell surface bound Notch and CeN molecules in third instar wing discs (Figures 6B–6D and S10; see Materials and Methods for details). In these experiments we observe internalization and change of subcellular localization of labelled Notch and CeN molecules within 10 min of labelling, suggesting that this traffic is likely to be an active process. Altogether these observations suggest that the CeN and endogenous Notch proteins are actively endocytosed.

There are no clear endocytic motifs in the intracellular domain of Notch. However, deletion of the RAM-ANK domain (CeN-ΔRANK) leads to the accumulation of the protein in the apical region of the cell (Figure S11B), either in the cell surface or in early endosomes, indicating that this domain is not necessary for the export of the molecule to the cell surface but that it is important for its endocytosis and traffic. This finding is highlighted by specific mutations in the ANK domain: receptors with point mutations in the ANK repeats, CeN-DM1, and CeN-DM2 (see Materials and Methods), also accumulate in the apical region of the cell near the adherens junctions and are not properly internalized (Figure S11C and S11D). An uptake experiment in wing discs expressing CeN-DM1 clearly shows that this mutant form has an impaired traffic, probably slower (Figure S12). In contrast to CeN, which causes gain of function phenotypes, these mutant proteins have no activity when overexpressed in the imaginal discs on their own, i.e., they produce no visible phenotype (unpublished data). These results suggest that the activity of CeN requires its traffic and not cleavage or nuclear translocation.

Altogether these results suggest that Notch undergoes very effective traffic in a ligand-independent manner and that endocytosis and traffic depends on structural motifs located in the intracellular domain.

### Notch Associates with a Pool of Armadillo and Promotes Its Traffic from the Adherens Junctions

The phenotypes caused by the expression of Arm^S10^ in the absence of Notch lend support to the observation that both proteins interact and that in normal conditions Notch can downregulate both the amount and the activity of Arm [43],[48]–[50]. Our experiments further suggest that this downregulation is mediated through the traffic of Notch. If this is indeed the case, we should observe Notch and Armadillo associated in endosomal vesicles and we might expect that the overexpression of Notch should affect the distribution of Arm^S10^ as well as of endogenous Arm.

**Figure 7:**
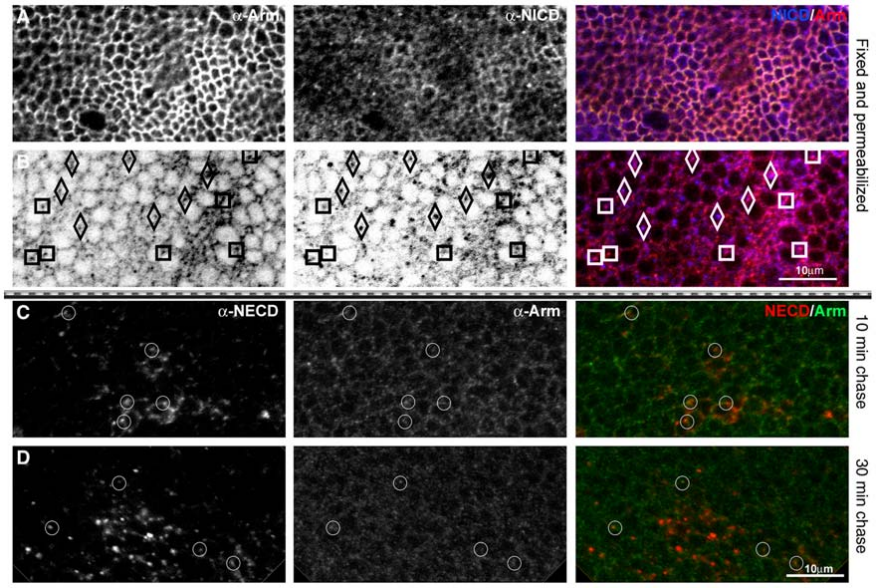
Colocalization of Notch and Arm in endocytic vesicles. (A) Notch and Armadillo colocalize at the apical membrane and in basally located vesicles. Image of a third instar wing disc of Arm-GFP flies in the region of the wing margin, fixed and stained for Arm (red channel), and NICD (blue channel). (A) Apical confocal section, at the level of the adherens junctions. (B) Basal section, 7 µm underneath the apical section. There is a fair amount of colocalization and a preliminary analysis of the colocalizing vesicles indicates that there are two types: those in which there is more Arm than NICD staining (marked with squares in the figure), and those that seemed to have more Notch than Arm (diamonds in the figure). Scale bar, 10 µm. (C–D) Results from an anti-Notch antibody loading and chase in wing discs expressing FLN under the control of *dpp*-Gal4. The antibody is against the extracellular domain of Notch (red channel) chasing for 10 min (C), and 30 min (D). The same discs were stained for Arm (green channel) after fixation. Note that there are Notch vesicles colocalizing with Arm, suggesting that both molecules traffic together. Subapical sections from the dorsal wing pouch are shown. Circles mark some of the NECD, and Arm colocalizing vesicles. Scale bar, 10 µm.

**Figure 8:**
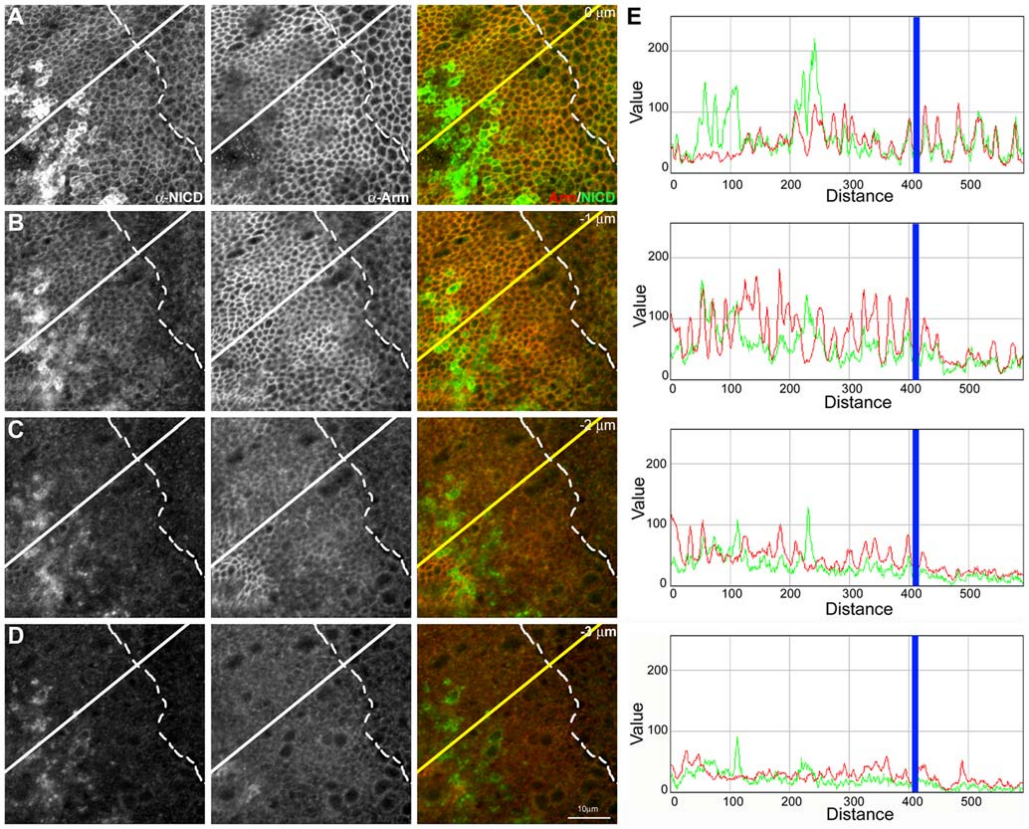
Notch recruits Armadillo to an apical domain in epithelial cells. (A–D) Sequence of confocal sections (1 µm apart) from a third instar wing disc expressing FLN in the *dpp* domain of expression (located left of the dashed line; for details see Materials and Methods). Note that overexpression of FLN promotes the expansion of the cell surface–located Notch as revealed by anti-NICD antibody (green channel) to two sections (A and B) spanning at least 2 µm, rather than the single one in the adjacent wild-type cells. This expansion is mirrored in the localization of Armadillo (red channel), confirming the interaction between Notch and Armadillo and establishing the fact that FLN is able to recruit endogenous Armadillo to its specific apical domain. In (C and D) it is possible to observe an accumulation of Armadillo (both diffuse and in vesicles) also correlated with the presence of FLN. Scale bar, 10 µm. (E) Fluorescence intensity profiles of α-NICD (green line) and α-Arm staining (red line) along the yellow line in the (A–D) confocal sections. The blue line shows AP boundary (for details see Materials and Methods). Note that there is a clear increase in the Arm levels where there is overexpression of FLN (left from the blue line), that is more obvious in the two first (apical) sections.

**Figure 9:**
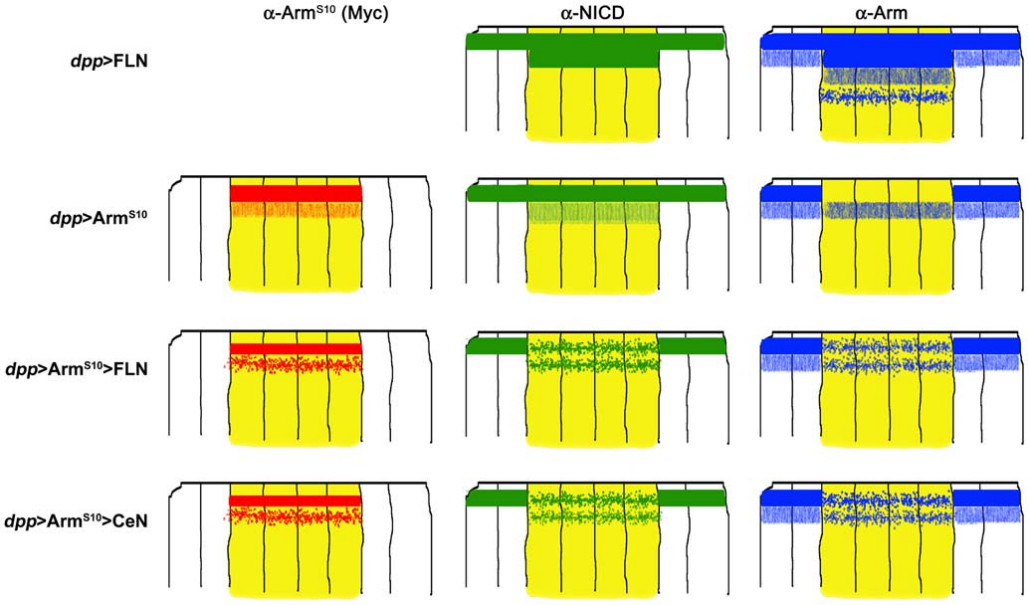
Schematic summary of the reciprocal effects of Notch and Arm on their relative subcellular localization. Each set of drawings represents a transversal section through the central region of a wing disc, in which cells expressing the indicated constructs are in yellow (wild-type ones are shown in white). The localization of the Myc-tag (from the Arm^S10^ molecule) is in red; NICD, in green; and the endogenous Arm in blue. See legends of Figures 8 and S13 for details. Expression of FLN shows that Notch can recruit Arm to its apical domain and also create a subapical domain where both can be found, sometimes, in vesicles. Arm^S10^ displaces endogenous Arm from the adherens junctions into a subapical domain, which is probably due to the increased stability of Arm^S10^. Expression of FLN or CeN together with Arm^S10^ leads to changes in the localization of Notch, Arm, and Arm^S10^ as shown. Overexpression of FLN shows that Notch can interact with Armadillo, which is in agreement with previous observations [43]. On the other hand, when FLN is overexpressed with Arm^S10^, it induces changes in the localization of Arm^S10^ and Arm. While it is likely that Notch can interact with all forms of Arm, it is also possible that it interacts preferentially with Arm^S10^ and that the effects that we observe on Arm under these conditions are the result of the interactions with Arm^S10^. The observation that in the presence of FLN and Arm^S10^, Arm can be observed at the adherens junctions favours this possibility. NB: Most of the effects that we observe are restricted to the apical and subapical domains of the epithelial cells and it is important to bear in mind that it is not easy to discern much structure in this domain at the level of light microscopy as this appears to be the location of early, mid, late, and recycling endosomes.

In fixed tissue we observe a high degree of colocalization between Notch and Armadillo in puncta that probably correspond to endocytic vesicles (Figure 7A and 7B). This finding is confirmed by the observation that in antibody uptake and chase experiments, it is possible to observe some of the endocytosed Notch vesicles associated with Armadillo (Figure 7C and 7D). The ability of Notch to interact with and possibly to recruit Armadillo is further demonstrated by the observation that overexpression of a full length Notch molecule in wild-type cells leads to an expansion of the domain of Armadillo localization to a broader apical domain with a subapical vesicular pool within the domain of Notch overexpression (Figures 8 and S13). In the case of Arm^S10^, analysis of the effect of Notch on Arm must take into consideration the effects of Arm^S10^ on endogenous Arm, which is displaced from the adherens junctions into a cytoplasmic pool [43]. Full length Notch reduces cell surface Arm^S10^, which can now be found in a large pool of subapical vesicles, and increases the number of apical vesicles of the endogenous Arm within its domain of expression. The alterations induced by Notch (summarized in Figure 9) correlate with a decrease in the concentration and activity of Arm observed before [43].

**Figure 10:**
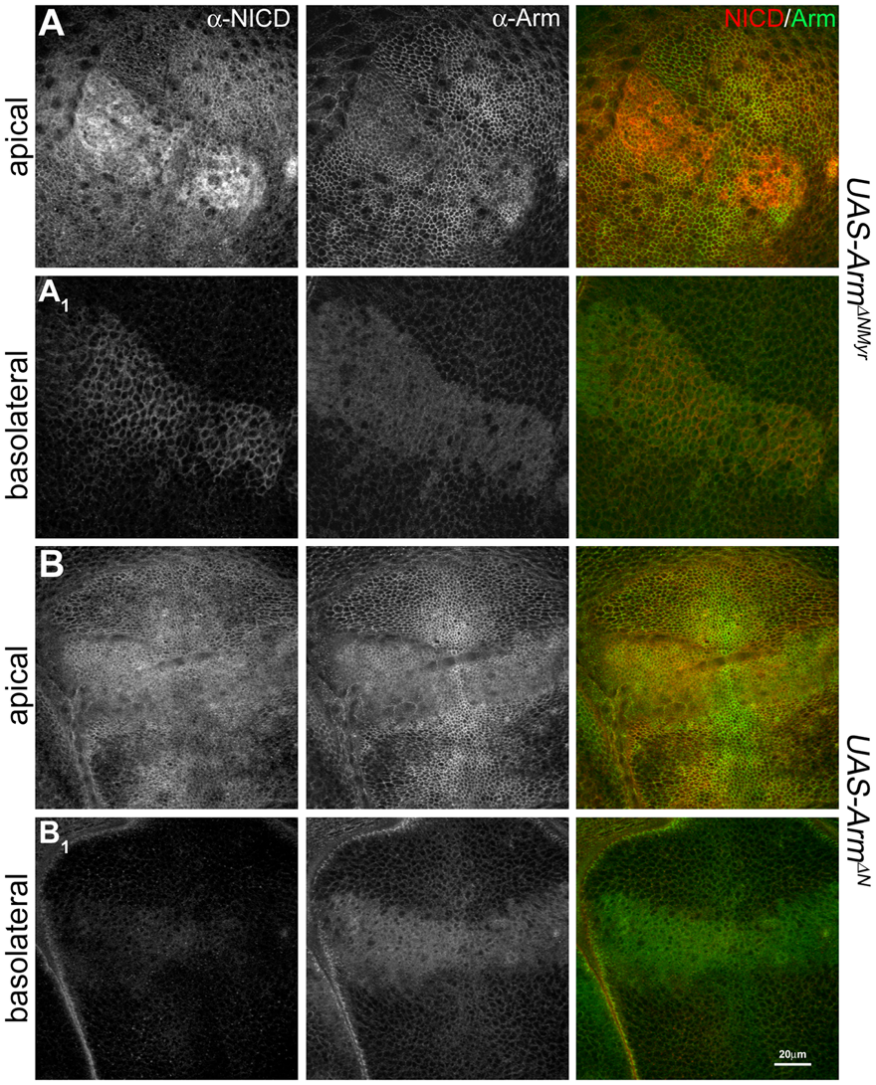
Membrane tethered activated Arm induces relocalization of Notch in epithelial cells. Analysis of the distribution and localization of Notch, monitored with a NICD antibody (in red), and endogenous Arm (in green) in wing discs expressing a myristylated N-terminal deleted Armadillo, Arm^ΔNMyr^ (A), or the same mutant without the myristylated signal, Arm^ΔN^ (B), under the control of *dpp*-Gal4. Confocal sections of the wing pouch are shown at the level of the adherens junctions (A and B) and a more basolateral (A_1_ and B_1_) region. The expression of Arm^ΔNMyr^ promotes an obvious accumulation of endogenous Notch in the apical (A) and basolateral (A_1_) membranes of the cells. The effect is very mild, though still visible, in the case of Arm^ΔN^. Both forms of Arm also cause an effect in the endogenous Arm: it is weakly displaced from the apical membrane and accumulates in the cells, more obvious in Arm^ΔN^ (B_1_, for details of the distribution, effects, and interactions of these mutants with endogenous Armadillo, see [70]). Scale bar, 20 µm.

The effects of Notch on Arm are mirrored by the effects of Arm on Notch: expression of Arm^S10^ induces a delocalization of Notch and CeN from the cell surface into a diffuse subapical domain and a general reduction in the amount of Notch or CeN in the cell (Figure S13B, S13C, S13B_1_, and S13C_1_). We interpret these observations as resulting from the titration of molecules involved in the regulation of Wnt signalling by the very stable Arm^S10^ [67],[68], which in our case leads to a concomitant alteration in the localization and traffic of Notch. Expression of a different form of activated Arm, (Arm^ΔNMyr^), an N-terminally deleted myristylated form [69] emphasizes these interactions: this form of Arm distributes itself throughout the membranes of the cells [70] and induces a relocalization and concentration of Notch to the sites of Arm^ΔNMyr^ expression (Figure 10A). The accumulation that we observe is likely to result from the removal of Notch from its normal sites of traffic and degradation. A similar form of Arm lacking the membrane association (Arm^ΔN^) exhibits a much weaker interaction with Notch (Figure 10B), underscoring that the pool of Notch that Arm interacts with is membrane bound.

Altogether these observations support an interaction between Notch and Armadillo, and show that Notch downregulates, in a ligand- and Su(H)-independent manner, the activity of Arm by changing its localization to vesicles where it may be sequestered or targeted for degradation. This finding is corroborated by the observation that CeN can suppress the ability of Arm^S10^ to activate Wnt signalling in a dominant manner (unpublished data) as we had shown before for the related molecule TN [43].

## Discussion

**Figure 11:**
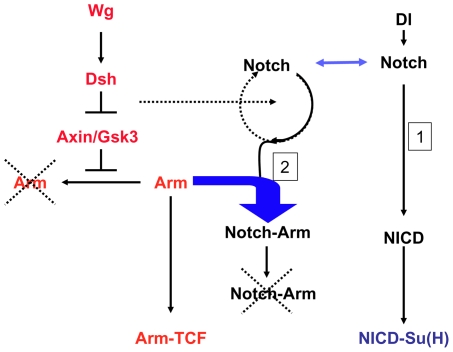
Mechanism for the buffering activity of Notch on Armadillo. In the absence of Wingless (Wg), Axin/Gsk3-based destruction complex degrades Armadillo. In the absence of Wg, Dsh inhibits the complex, and Arm can enter the nucleus. We postulate that Notch is endocytosed through two different routes, one ligand dependent, which leads to the generation of NICD and Su(H)-dependent signalling and the other, ligand independent (2), which leads to degradation. In the ligand-independent route Notch associates with Armadillo/ß-catenin and directs it to degradation. This ligand independent activity of Notch would degrade the Arm that escapes from the Axin-mediated degradation.

There is evidence that the Notch receptor can downregulate the activity of Arm/ß-catenin and that this modulation is important in the homeostasis of Wnt signalling [43],[45],[48]–[50]. Here we begin to explore the mechanism of this interaction and find that the effect of Notch on Wnt signalling is mediated by the ligand-independent traffic of the Notch receptor. Our results show that in addition to its role as a membrane-tethered transcription factor [4],[15],[71], Notch has a second activity associated with its constitutive endocytosis and traffic, which is used to target the amount and the activity of Armadillo (see Figure 11 for details). Since this function of Notch has been established under experimental conditions that require simultaneous loss of function of Notch and gain of function of Armadillo, it is reasonable to ask if it plays a role during normal development. We believe that it does. There is an ample literature of interactions between Notch and Wnt signalling that do not admit simple explanations in terms of their joint transcriptional activities (reviewed in [3]), and which can be accounted for by our findings here. Furthermore the interactions between Notch and Armadillo during the traffic of the receptor provide a mechanistic framework to interpret the observations that loss of function of Notch leads to activation of ß-catenin [38],[43],[50] and the existence of a class of gain of function alleles of Notch that modulate Wnt signalling independently of Su(H), but in a GSK3 dependent manner [72],[73]. The context dependence of these observations are likely to reflect tissue-specific inputs of the machinery of traffic on Notch. Taking into account these reports and the results presented here we favour the possibility that the function of Notch we have uncovered does function to buffer the activity of Armadillo in vivo. Our finding not only provides a mechanism for the interactions between Notch and Wnt signalling but also explains the phenotypic differences associated with the loss of function of Notch and that of its bona fide ligands and effector. It also opens the possibility that molecules other than Armadillo that interact with its intracellular domain are modulated by Notch in a similar manner.

### Notch as a Buffer for the Activity of Armadillo

The relationship we have uncovered between Notch and Armadillo in *Drosophila* is reminiscent of that described in the skin of mice where targeted removal of *Notch1* results in high levels of activated ß-catenin that prime the cells for the development of tumours [38],[39]. In both, the wing disc and the skin, the defects ensue from two sequential steps: loss of a tumour suppressor (*Notch*) followed by activation of an oncogene (*Armadillo/*ß*-catenin*), which sensitizes the system for the development of tumours. This sequence is well characterized in human cancers and our results suggest that *Drosophila* could be a good experimental system to study its causes and possible therapies. In the imaginal discs, this activity of Notch, which has been proposed to set a threshold for Wnt signalling [3],[43], can modulate growth and patterning in the rapidly dividing epithelium and might provide a paradigm for similar interactions and function in other systems.

The large aggregates of cells that result from the activation of Armadillo in the absence of Notch could be construed as tumours, as they exhibit overgrowth and defects in polarity and adhesion. However, this correspondence awaits further experiments, and they might correspond to cells with compromised differentiation. Whatever the nature of these aggregates, this activity of Notch is not restricted to the developing wing as the same effect is observed in all imaginal discs (unpublished data). As on their own neither increased Armadillo activity nor loss of Notch function elicit a similar effect, these cells represent a synthetic phenotype that reveals the physiological potential of these pathways as well as their close interactions.

The effects of loss of Notch function in the mouse skin and the imaginal discs show that Notch performs an important function as a buffer against fluctuations in the activity of Arm/ß-catenin, and that as such it plays a role in the homeostasis of the cell. It is likely that the Axin/APC/GSK3-based complex that degrades Armadillo and ß-catenin is not totally effective and that, even in the absence of Wnt ligands, there are leakages of active Arm/ß-catenin that result in small bursts of signalling. We surmise that the role of Notch is to interact with the leaked activated Arm/ß-catenin and to degrade it in order to maintain the levels of spontaneous signalling low, thus providing its buffering function. The existence of complexes between Notch and Armadillo has been reported before [43]–[47] and is supported by our observation of their reciprocal change of localization in the overexpression experiments. There is little question that some of these interactions are likely to be associated with the transcriptional activity of both molecules, but our observations that Notch is able to recruit Armadillo to an apical domain, that endocytosed Notch can be found associated with Armadillo and that Arm^S10^ is stabilized in the absence of Notch, provide evidence for another level of interaction that is likely to be the basis for its buffering activity. This function might be associated with features of tumour suppressors as it would provide the mechanism to cope with transient high fluctuations in the amount or activity of oncogenes. It also raises the possibility of an association between the levels of Notch and the oncogenicity of ß-catenin, i.e., there might be a tissue specific traffic of Notch that determines its tumour sensitivity.

Our observations also have implications for the mechanism of activation of Arm/ß-catenin. There is evidence for distinct pools of Arm/ß-catenin involved in signalling and adhesion and, although it is generally accepted that the signalling pool is associated with a cytoplasmic soluble pool, a number of experiments cast doubts on this simple relationship [74]–[79]. Our results support the existence of an active pool of Arm/ß-catenin that, in epithelial cells, exists at or near the adherens junctions, and that it is this pool, rather than the general GSK3-sensitive pool, that is the target of Notch (see also [43]). A GSK3-insensitive pool subject to further regulation by Axin has been suggested as the source of active Armadillo [67],[79], and interactions have been described between Axin and Notch in the regulation of Armadillo [48]. It might be that this pool corresponds to the membrane-associated pool that we identify here and that rather than a putative cytosolic pool, it is this pool that contains the transcriptionally competent Arm/ß-catenin. On the basis of these observations we surmise that, in the absence of a Wnt signal, Notch sequesters a cell surface-located pool of Armadillo, probably active Armadillo, traffics with it, and induces its degradation. This possibility is consistent with the effects of overexpression of Notch on the amount and localization of Arm^S10^ (Figures 8, 9, and S13) and with the observation that suppression of endocytosis and traffic by mutations in the *Drosophila* Dynamin encoded by *shibire* result in a substantial increase in the amount of Armadillo ([80] and unpublished data).

Our results argue for a function of the traffic of Notch in the regulation of the activity and the amounts of Armadillo. However, in the mouse skin, Nintra can suppress some of the tumorous phenotypes caused by loss of *Notch1* function by modulating the activity and the amount of ß-catenin [38],[81],[82], and similar interactions have been observed in other systems [81],[82]. Although it is possible that this reflects a contribution of the transcriptional activity of Notch to the regulation of ß-catenin, we think this is unlikely to provide the major component as the suppression of ß-catenin is not Su(H) dependent [49], and in some cases the effect is not only on the activity of ß-catenin but also its amount [38]. Furthermore, there is increasing evidence that Nintra can perform activities and interactions that are not easy to reconcile with its function as a transcription factor [83]–[86]. One possibility is that cleaved Nintra has interactions and activities that do not involve Su(H) and its vertebrate counterparts, but it might also be the case that under experimental conditions in which there is an excess of this form of Notch, some of it engages in abnormal complexes with proteins that the intracellular domain of Notch normally interacts with, triggering a squelching effect [87] with functional consequences. Taking into account all the evidence presented here, we believe that squelching might be the cause of many of the interactions reported between Nintra and Armadillo/ß-catenin. It will be important to bear this in mind when interpreting the effects of overexpressing Nintra, particularly in cultured systems.

### Wnt-Notch (Wntch): Signalling and Trafficking

It could be argued that the effects of CeN and related molecules are due to “neomorphic” effects. We believe that this is not the case. In fixed-tissue and antibody uptake and chase experiments, a fraction of CeN colocalizes with Notch, suggesting parallel activities of the two molecules. It is likely that the effects of CeN reveal the strong dominant effect on Su(H)-independent activities of Notch, much like Nintra reflects the transcriptional activity of the receptor. CeN also points to the existence of a pool of Notch that is usually in limiting amounts but active in specific trafficking functions. Thus we believe that the activity of CeN reveals the ligand-independent activity of the Notch receptor that targets the activity of Armadillo, and which is mediated by a pool of receptor that is not engaged in Delta, Serrate, Lag1 (DSL)–dependent signalling.

Interestingly, Wnt signalling itself affects the traffic of Notch and promotes the degradation of the ligand-independent forms we use in our experiments (unpublished data, manuscript in preparation). This observation is consistent with the notion that Wnt signalling promotes the degradation of molecules that oppose its activity, e.g., Axin [79],[88], and this includes Notch (unpublished data, manuscript in preparation). Furthermore, it is likely that the interaction between Dishevelled and Notch that has been described [41],[43],[45] is part of this regulatory interaction. Altogether these and the increasing number of reports of structural and functional interactions between elements of these two pathways lend support to the notion that both act in an integrated manner as a single functional module, which we have dubbed Wntch (for Wnt and Notch signalling) [3].

Our observations and conclusions could account for the frequent appearance of defects in Notch traffic and signalling in screens geared to uncover tumour suppressors in *Drosophila* [24],[32],[89],[90]. We would like to suggest that Notch might be used to link the endocytic pathway and traffic apparatus to integrate and modulate signalling events, a function that might play crucial roles in the development of organisms and particularly in tissue homeostasis. A corollary of this suggestion is that the strong requirement for endocytosis and traffic in the transcriptional activity of Notch might be associated with its role in trafficking, which might be evolutionarily ancestral to its role as a transcription factor and perhaps extend to elements of signalling pathways other than Wnt.

## Materials and Methods

### Genetics

The following *Drosophila* UAS and Gal4 stocks were used: (w;;*dpp*-Gal4/TM6B); (w;UAS-CeN/CyO^ftz^;MKRS/TM6B); (w;UAS-CeN-DM1/CyO^ftz^;MKRS/TM6B); (w;UAS-CeN-DM2/CyO;MKRS/TM6B); (w;UAS-CeN-ΔANK/CyO;MKRS/TM6B); (UAS-Arm^S10^ on the 2^nd^); (UAS-Arm^S10^ on the X); (UAS-Ce); (UAS-CN); (w; If/CyO^wg^;UAS-FLN); (UAS-Arm^ΔNMyr^); (UAS-Arm^ΔN^ a gift from G. Struhl); (Arm-GFP).

To generate the clones using the FRT/FLP system, (Df(1)N^81k1^ [FRT101w+]/FM6; ; dpp Gal4, UAS FLP/TM2) females were crossed to (ywGFP [FRT101w+]/Y; UAS-Arm^S10^), (ywGFP [FRT101w+]/Y; UAS-Arm^S10^/CyO; UAS-FLN/TM6B), (ywGFP [FRT101w+]/Y; UAS CeN, UAS-Arm^S10^/CyO^ftz^), or (ywGFP [FRT101w+]; UAS Arm^S10^; UAS Nintra/SM6a-TM6B) males.

To generate Notch clones using the MARCM system, N^55e11^FRT19A/FM7-GFP, N^55e11^FRT19A/FM6; UAS-Arm^S10^ or N^55e11^FRT19A/FM6; UAS-Arm^S10^, CeN females were crossed to P{ry[+] neoFRT19A}19A, P{w[+] tubP-GAL80} L1, P{ry[+] hsFLP}1, w; CyO/P{w[+] UAS-nucZ}20b, P{w[+] UAS-CD8:GFP} LL5; TM6, Tb, Hu/P{w[+] tubP-GAL4} LL7 males (FRT19 MARCM stock). Clones were induced in larvae 24–48 h, 48–72 h, or 72–96 h AEL by applying a 1-h heat shock (37°C). To generate the control clones (FRT19A;UAS-CeN/+), (FRT19A;UAS-Arm^S10^/+), or FRT19 males were crossed to females from the FRT19 MARCM stock and induced similarly in 48–72-h and 72–96-h AEL larvae.

To generate the ligand mutant clones, (UAS-Arm^S10^/+; FRT82, Dl^rev10^, Ser^Rx82^/+) or (FRT82, Dl^rev10^, Ser^Rx82^/TM6B) males were crossed to (hsFLP,tub-Gal4,UAS-GFP/FM6;;FRT82,Tub-Gal80/TM6) females induced similarly in 48–72-h AEL larvae. For the Su(H) clones (UAS-Arm^S10^;FRT40, Su(H)^Δ47^/+) or (w;FRT40, Su(H)^Δ47^/CyO) males were crossed to (hsFLP;FRT40,Tub-Gal80;tub-Gal4,UAS-GFP/SM6-TM6B) females induced similarly in 48–72-h AEL larvae. The clones expressing Arm^S10^ were recognized by α-Myc staining.

### Molecular Biology

CeN-DM1 and CeN-DM2 were generated by PCR-based mutagenesis of the sequence encoding the intracellular domain of Notch (amino acids 1767–2703) and subcloning of the resulting constructs into the pUAST-DEST12 vector.

UAS-CN: The sequence-encoding extracellular and transmembrane domains of CD8 (obtained from CeN-DM1 construct) was cloned into pUAST using the KpnI and XbaI sites to generate UAS-CD8. The reverse primer used for amplification of the CD8 fragment contained a MluI site in addition to the XbaI site. The NICD sequence was amplified from pENTR-NICD and cloned into UAS-CD8 using the MluI and XbaI sites.

UAS-Ce: The CD8-eGFP sequence was amplified from UAS-CeN-DM1 and cloned into the KpnI and XbaI sites of pUAST.

UAS-CeN was generated from the UAS-CeN-DM1 mutant construct. UAS-CeN-DM1 was digested with BsiWI to remove the fragment of DNA containing the DM1 mutation. pENTR-NICD was also digested with BsiWI to generate the equivalent wild-type NICD fragment. This wild-type fragment was ligated into the remainder of the BsiWI-digested UAS-CeN-DM1 plasmid to replace the mutated version. Correct insert orientation was ascertained by digestion with MfeI and BsiWI.

UAS-CeN-ΔRANK: The DNA sequence encoding residues 2142–2703 of *Drosophila Notch* was amplified from UAS-FLN and cloned into the XbaI site of UAS-Ce. Correct insert orientation of the resulting clones was assessed using MfeI and BsiWI.

### Immunohistochemistry

The antibodies used in this study were: mouse monoclonal antibody against the extracellular domain of Notch, α-NECD, (C458.2H, 1∶5, DSHB); rat monoclonal against E-Cadherin (DCAD2, 1∶20, DSHB); antibody against the intracellular domain of Notch, α-NICD (mouse monoclonal C17.9C6, 1∶10, DSHB; and sheep antisera generated in the lab, 1∶1,000); α-senseless (from H. Bellen); α-Armadillo (N27A1, 1∶20, DSHB; and rabbit antisera 1∶50, from A. Muller); α-Scribble (1∶1,000; from C. Doe); α-Myc (1∶1,000; from AbCam); Rab7 and Sara (1∶100, from M. Gonzalez-Gaitan); Carnation (1∶750, from H. Krämer); Alexa-conjugated secondary Ab (1∶200) from Molecular probes.

### Fixed Tissue Stainings

Imaginal wing disc were dissected from third instar larvae and fixed for 30 min (4% paraformaldehyde in balanced salt solution [BBS] with 1 mM CaCl2). Then they were immunostained with the indicated antibodies in BBS (50 mM BES, 280 mM NaCl, 1.5 mM Na2HPO4.2H2O)+0.1% Triton X-100, 0.5% BSA 1 mM CaCl2) using standard antibody staining protocols. Discs were mounted in Vectashield and viewed using a confocal microscope (see below).

### Pulse-Chases

Imaginal wing disc were dissected from third instar larvae in cold BBS. Discs were pulse labelled with mouse α-NECD (a 1∶2 mix of C458.2H DSHB supernatant in BBS) and/or α-CD8 (1∶15 from Caltag Laboratories) for 1 h at 4°C. After washing six times for 2 min each in cold BBS at 4°C, the discs were either fixed immediately (0-min chase) or transferred to Clone 8 medium at 19°C for 10, 30, or 60 min. Fixation was done in 4% paraformaldehyde (in BBS) at room temperature for 30 min. Afterwards standard antibody staining protocols were used.

Comparing the results of both protocols, we got the impression that the antibodies used on fixed tissue reveal the most stable pool of protein, while the pulse-chases reveal a specific pool that shows where the protein is located in that moment.

### Image Analysis

Wing discs were examined under a Nikon Eclipse E800 microscope coupled to a BioRad MRC1024 or Zeiss LSM 510-Meta confocal units. The images of the pulsed-chased wing discs of the different time points were acquired in the same conditions of laser intensity, gain, and pinhole, and processed exactly the same way. Adobe Photoshop and Excel were used to assemble the figures and to quantify the clone areas in pixels. For the analysis of the relative size of the clones in different genetic backgrounds, images from third instar imaginal discs were assembled at the same resolution and magnification. Clones in chosen regions were then highlighted with a lasso and their areas calculated in pixels using Photoshop toolkit and Excel. The fluorescent intensity profiles were performed with the software package ImageJ (RGB Profiler plugin).

## Supporting Information

Figure S1 **Armadillo induces growth and ectopic neurogenesis in the absence of Notch activity.** (A) Wing disc with clones of Notch mutant cells (marked by the absence of GFP, green channel) along the AP boundary generated using the FRT/FLP system. The clones are small, consistent with low growth rate and apoptosis [52],[91]. (B) Wing disc expressing UAS-Arm^S10^ under the control of *dpp*-Gal4. Notice the change of morphology that is related to an extension of the hinge (asterisk) and to a small ectopic wing pouch in the scutellar region (white arrow). (C) Wing disc with clones of *Notch* mutant cells expressing UAS-Arm^S10^ under the control of *dpp*-Gal4 (see Materials and Methods for details). Notice that in contrast with (A), the clones are large and also the disc is larger. The comparison of these with the MARCM clones suggests that the ones generated with the FRT/FLP system represent all the stages in the formation of the outgrowths. The images in (A–C) are in the same magnification; the dashed line indicates the AP boundary. The blue channel in (C) shows Arm^S10^ expression (using α-Myc antibody). (A–C) were taken at the same magnification. (D–F) Higher magnification images of prospective nota showing the expression of Senseless, which labels cells in proneural clusters and represents a high threshold target of Wingless signalling (red channel). (D) In *Notch* mutant clones, cells within the realm of proneural clusters express Senseless reflecting a failure in lateral inhibition and a high activity of Wingless. (E) Expression of Arm^S10^ in the notum does not elicit ectopic neural expression. This is in contrast with its effect in the wing pouch where it always elicits ectopic neural expression [43]. (F) In the absence of *Notch* Arm^S10^ elicits ectopic expression of Senseless in the notum outside the proneural clusters domain (compare to [D]).
(6.84 MB TIF)

Figure S2 **In the absence of Notch, Armadillo induces defects in cell proliferation, adhesion, and polarity at all stages of disc development.** Clones induced at 24–48 h of development. Confocal images of third instar wings discs with MARCM clones of *Notch* mutant cells (labelled in green) that overexpress Arm^S10^ induced at 24–48 h AEL. (A) is apical, and (A_1_) is a basolateral section. The red channel shows Scribble (a basolateral cell junction marker). The very dense single sphere of cells is characteristic of these clones and appears to have been engulfed by wild-type cells, which wrap around them. Most of the cells in the sphere have an abnormal polarity as revealed by the loss of Scribble (yellow arrow in [A]). The yellow continuous line marks the position of the clone, in (A_1_) is interrupted to show clearly the change of polarity of those cells (yellow arrow in [A_1_]). The complete z-stack of this clone can be found as Video S1. Scale bar, 20 µm.
(6.62 MB TIF)

Figure S3 **Effect of Notch on the activity of Armadillo in clones of cells of different ages.** Confocal images of third instar wings discs with MARCM clones of *Notch* mutant cells (labelled in green) that overexpress Arm^S10^ without (A and B) and with (C and D) CeN, induced at 72–96 h (A and C), 48–72 h (B and D). (A–D) are apical, and A_1_–D_1_ are basal sections. As in Figure S2, the red channel shows Scribble (a basolateral cell junction marker) and as in other figures, the pictures on the top and the right represent optical z-section through the clones following the green and the red lines shown in the main picture. The small insets at the corners of each image are low magnification pictures of the discs shown, which act as a reference. Note that the size and appearance of the clones change depending on the stage of the induction: the late induced clones are smaller and exhibit an irregular shape (A), while the early ones are bigger and rounder (B). Often the clones lose the basal connexion (white arrow in [A]), some can be seen to coalesce (with one in the peripodial membrane in [B], white arrowheads), and some cells within the clones lose their polarity (blue arrows in [B]). The clones in the wing disc cells are depicted with a continuous line, and those in the peripodial membrane in a dashed line. Expression of CeN rescues the effect of the loss of function of Notch on the activity of Arm^S10^ (C and D). These clones are smaller, they do not fuse, recover their polarity and span the epithelium. The complete z-stack of (B) and (D) can be found as Videos S2 and S3, respectively. Scale bar, 20 µm.
(6.72 MB TIF)

Figure S4 **Wing imaginal discs with clones as shown in** **Figure 2** **.** Confocal images of third instar wings discs with MARCM clones (labelled in green) of *Notch* mutant cells (A and A_1_), of *Notch* mutant cells that overexpress Arm^S10^ (B and B_1_), and of *Notch* mutant cells that overexpress Arm^S10^ and CeN (C and C_1_), induced at 48–72 h (A–C) or 72–96 h AEL (A_1_–C_1_). Notice that the clones expressing Arm^S10^ are much larger and display a rounded appearance. The red channel shows Scribble, the clones are labelled in green. This image corresponds to the discs shown in the inset of Figure 2 and allows a magnified visualization of the distribution and shape of the clones in the imaginal discs. Scale bar in (C_1_), 100 µm.
(5.91 MB TIF)

Figure S5 **MARCM clones of cells with different genotypes.** Confocal images of third instar wings discs with MARCM clones of wild-type cells (induced in the FRT19 background, see Materials and Methods for details) (A and A_1_), Arm^S10^ (B and B_1_), or CeN (C and C_1_) (labelled in green) induced at 48–72 h AEL (A–C) 72–96 h AEL (A_1_–C_1_). The wild-type clones exhibit the well-described appearance with an elongation across the dorsal-ventral (DV) axis. Clones of cells expression Arm^S10^ do not exhibit increased growth, though they appear smaller and a bit more rounded. They still maintain the apico-basal polarity (compare with the effects of expressing Arm^S10^ in the absence of Notch, e.g., Figures 2 and S7). The Myc label (red) highlights the nuclear localization of Arm^S10^ under these conditions. The clones of cells expressing CeN are smaller than wild type and show a tendency to be more rounded. The blue channel shows the DCadherin staining; the red is α-Myc in B and B_1_, and α-NICD in C and C_1_. Scale bar, 20 µm.
(10.00 MB TIF)

Figure S6 **Activated Armadillo is stabilized in the absence of Notch.** Confocal images of third instar wings discs expressing Arm^S10^ in clones in the presence (A) and absence (B) of Notch. (A) Wing disc with a clone of cells that overexpress Arm^S10^ (labelled in green) induced at 72–96 h AEL. (B) Wing disc with a MARCM clone of *Notch* mutant cells that overexpress Arm^S10^ (labelled in green) induced at 72–96 h AEL. The blue channel shows Arm^S10^ expression (using α-Myc antibody), the green highlights the membrane and the cortex. Both images were taken under the same confocal conditions at the level of the nuclei. Note that in the absence of Notch there are increased levels of Arm^S10^, which now can be observed prominently in the nuclei (arrows). A small region of the nucleus is devoid of staining in some sections (arrowhead); this region probably corresponds to the nucleolus. Scale bar, 10 µm.
(3.97 MB TIF)

Figure S7 **Comparison of the Notch and Su(H) mutant clones with and without expression of Arm^S10^.** Confocal images of third instar wings discs with clones of cells induced between 72–96 h AEL mutant for both *Notch^55e11^* (A and C) or *Su(H)* ^Δ*47*^ (B and D), without and with Arm^S10^ as indicated (labelled in green). This late generated *Su(H)* ^Δ*47*^ clones of cells already have apoptotic cells on the basal side (white arrows), which are not seen in the *Notch^55e11^*. Expression of Arm^S10^ in these cells increases the size of the *Su(H)* mutant clones, alters their appearance, and reduces, but does not eliminate, the number of apoptotic cells in the basal region or the interdispersion of the clones. In the *Notch* mutant clones, the expression of Arm^S10^ increases the size of them and there are fewer (probably due to the coalescence of them) Technical details of the images as in Figure 2 with the small insets at the corners of each image are low magnification pictures of the discs shown which act as a reference. The complete z-stack of (B) and (D) can be found as Videos S4 and S5. Scale bar in (D_2_), 20 µm.
(9.62 MB TIF)

Figure S8 **Effects of Nintra on the activity of Arm^S10^ in the absence of Notch.** Third larval instar imaginal disc expressing UAS-Arm^S10^ and Nintra under the control of *dpp*-Gal4, and with clones of cells mutant for *N^81k1^* generated using the FRT/FLP in the same manner as those in Figures 1 and 5 (and see Materials and Methods for details). (A) Image taken with a 10× objective; (B) higher magnification showing details of the clones. A comparison with Figure 5 shows that Nintra rescues the size of the clones. The discs are very large and elongated in the dorsal-ventral (DV) direction due to the effects of the interaction between Nintra and Arm^S10^ in the induction of the primordium [53]. It is worth pointing out that this interaction is amplified in this genetic background in which there is only one dose of Notch. It is also important to mention that the well-established interaction between Nintra and Arm cannot explain the suppression of the activity of Arm, which we observe in the clones. We surmise that this inhibition is mediated by squelching of Arm by the excess Nintra (see text for further details).
(4.56 MB TIF)

Figure S9 **Notch colocalizes with some endosomal markers.** Image of third instar wing discs fixed and stained for NECD (green channel) and Rab7 (A), Carnation (B), or Sara (C) (red channel). (A) and (C) are subapical sections and (B) is 7 µm underneath the level of the adherens junctions. Note that there are some vesicles in which NECD and Rab7 colocalize, more with Carnation and even more (nearly all) with Sara. Scale bar, 10 µm. Circles highlight some of the vesicles with colocalized stain.
(6.80 MB TIF)

Figure S10 **Endocytosis and traffic of CeN and Notch (extended version of** **Figure 6** **).** (A–D) Notch and CD8 tracked over time after pulsing live wing discs expressing CeN with *dpp*-Gal4 with an antibody against the extracellular domain of Notch (red channel) and CD8 (blue channel), and chasing for 0 (A), 10 (B), 30 (C), and 60 min (D) (details in Materials and Methods). (A–D) are apical sections at the level of the adherens junctions level; (B_1_–D_1_) are subapical sections, 1 µm underneath; and (B_2_–D_2_) are basal sections, 7 µm underneath the apical ones. After 0 min of chasing, most of the labelled endogenous Notch and the expressed CD8 localize to the apical membrane of the cells (A) and there are no vesicles in subapical or basal levels (A–A_2_). After 10 min of chase, both Notch and CeN localize in apical and subapical dots that correspond to vesicles (B–B_2_). After 30 min, the endogenous Notch and CeN have been cleared almost completely from the apical membranes and can be found mostly in subapical vesicles and also now in the basal domain (C–C_2_). After 60 min of chase, there is no cell surface labelled and the endogenous Notch localizes in vesicles in apical, subapical, and basal levels; at this time point, CD8 also goes to apical vesicles, but mainly in the subapical and basal levels (D–D_2_). The overall levels have decreased. In all cases the apical and basal images were taken in equivalent levels in the dorsal region of the wing pouch. The GFP highlights the steady state CeN against the background of the dynamic experiment. Scale bar, 10 µm. Circles highlight colocalized stain.
(4.30 MB TIF)

Figure S11 **Mutations in the ANK domain impair Notch traffic.** Wing pouch images from third instar discs expressing CeN (A–A_2_), CeN-ΔRANK (B–B_2_), CeN-DM1. (C–C_2_) and CeN-DM2 (D–D_2_) under the control of *dpp*-Gal4. All are derivatives of CeN; CeN-ΔRANK is a deletion of the RAM and ANK domains of the intracellular domain, whereas CeN-DM1 and CeN-DM2 are point mutations in the fourth and fifth ANK repeats (for details see Materials and Methods). All images were taken from the apical level of the wing pouch; the arrows point to the dorsal-ventral (DV) boundary; the eGFP fluorescence (from the CeN molecule) is shown in all cases. (A_2_–D_2_) are confocal optical z-sections through the wing pouches. Note that there is an apical accumulation of the CeN mutant molecules in apical levels, particularly clear in the CeN-ΔRANK and fewer vesicles in basal levels, which tend to be of larger size. These are images from a steady state, for pulse chase of one example see Figure S12.
(7.86 MB TIF)

Figure S12 **Endocytosis and traffic of CeN-DM1.** CeN-DM1 (see Figure S10) exhibits a Notch-related distribution and although it is endocytosed, its dynamics are much slower than CeN and than Notch, as revealed by the observation that in antibody uptake experiments it remains at the cell surface for longer than CeN. (A–D) Notch and CD8 (from CeN-DM1) tracked over time in the same cells by pulsing CeN-DM1 expressing live wing discs with an antibody against the extracellular domain of Notch (red channel) and CD8 (blue channel), and chasing for 0 (A), 10 (B), 30 (C), and 60 min (D). (A–D, apical; B_1_–D_1_, subapical sections; B_2_–D_2_, basal sections; these sections were taken at the same levels as the ones in Figure S10). After 0 min of chasing, the endogenous Notch and CD8 localize in the apical membrane of the cells (A) and there are no vesicles in subapical or basal levels (A–A_2_). After 10 min of chase, Notch and CeNDM1 can mainly still be found in the apical level (suggesting that it remains there for longer time), and some in subapical vesicles (B–B_2_). After 30 min, the endogenous Notch and CeNDM1 have been cleared almost completely from the apical membranes and can be found mostly in subapical vesicles that look bigger than the CeN ones. By this time the wild-type Notch and CeN (see Figure S10) can be detected in the basal domain but not CeN-DM1 (C–C_2_). After 60 min of chase, the endogenous Notch and CeN-DM1 localizes in apical, subapical, and basal levels; at this time point, CD8 also goes into vesicles, but mainly in the apical and subapical levels (D–D_2_). In all cases the apical and basal images were taken in equivalent levels in the dorsal region of the wing pouch. Notice the large amount of CeN-DM1 that is present at this stage, which contrasts with the lower levels of CeN (see Figure S10). Scale bar, 10 µm. Circles highlight colocalized stain. All the images of Figures 6, S10, and S11 were taken under the same confocal conditions and processed equivalently, so that we can compare levels of fluorescence. Comparing both CeN and CeN-DM1 localization at 0 and 10 min, it can be said that CeN-DM1 remains for longer time around the cell surface of the cell. At 30 min, there are bigger vesicles in CeN-DM1, suggesting again that this molecule has a defect in its traffic. Moreover, endogenous Notch and CeN (or CeN-DM1) colocalizing vesicles suggest that these molecules can traffic together through the cell. A comparison of this figure with Figure S10 suggests that CeN-DM1 can interfere with the traffic of endogenous Notch, as it appears that there is more Notch in the cell surface after 60 min than in the presence of CeN. However, this observation does not seem to have an effect on the function of Notch, as expression of UAS CeN-DM1 under several GAL4 drivers has no phenotypic effect in a wild-type background.
(4.91 MB TIF)

Figure S13 **Armadillo and Notch induce reciprocal alterations in their subcellular localization.** Analysis of the distribution and localization of Arm^S10^ (red channel), NICD (in green), and endogenous Arm (blue) in wing discs expressing UAS-Arm^S10^ (A); UAS-Arm^S10^;UAS-FLN (B); UAS-Arm^S10^,UAS-CeN (C), under the control of *dpp*-Gal4. Apical (A–C) and subapical (A_1_–C_1_; 1 µm below the apical section) confocal sections of the dorsal region of the wing pouch are shown. The expression of Arm^S10^ promotes an accumulation of endogenous Notch apically and subapically as revealed by increased diffused staining with anti-NICD antibody (A). This accumulation is probably due to the stability of Arm^S10^, which tends to reside in the apical region, apparently have a slower turn over, and thereby stabilize Notch in that region. This result can be seen apically and is particularly obvious subapically, where a large accumulation of Notch can be observed. Expression of both FLN and CeN with Arm^S10^ lead to changes in the distribution and appearance of both Arm^S10^ and endogenous Arm. Most significantly, overexpressed Notch induces a decrease in the amount of Arm^S10^ in the apical surface and its accumulation in the subapical in a diffuse form with some vesicles (compare [A] with [B] and [C]). This effect can also be seen in the endogenous Arm: Arm^S10^ displaces it from the apical membrane towards a subapical “shadow” that is gathered into vesicles by FLN and CeN both in apical and subapical sections (B–B_1_ and C–C_2_). Scale bar, 10 µm.
(9.77 MB TIF)

Video S1 **z-stack of confocal images of a third instar wings disc with MARCM clones of** ***Notch*** **mutant cells that overexpress Arm^S10^ (labelled in green) induced at 24–48 h AEL.** Red channel shows Scribble. This z-stack begins with the apical confocal sections.
(0.81 MB MOV)

Video S2 **z-stack of confocal images of a third instar wings disc with MARCM clones of** ***Notch*** **mutant cells that overexpress Arm^S10^ (labelled in green) induced at 48–72 h AEL.** Red channel shows Scribble. This z-stack begins with the basal confocal sections.
(1.76 MB MOV)

Video S3 **z-stack of confocal images of a third instar wings disc with MARCM clones of** ***Notch*** **mutant cells that overexpress Arm^S10^ and CeN (labelled in green) induced at 48–72 h AEL.** Red channel shows Scribble. This z-stack begins with the apical confocal sections.
(0.46 MB MOV)

Video S4 **z-stack of confocal images of a third instar wings discs with MARCM clones of** ***Su(H)*** **mutant cells (labelled in green) induced at 48–72 h AEL.** Blue channel shows DCadherin. This z-stack begins with the apical confocal sections.
(3.08 MB MOV)

Video S5 **z-stack of confocal images of a third instar wings disc with MARCM clones of** ***Su(H)*** **mutant cells overexpressing Arm ^S10^ (labelled in green) induced at 48–72 h AEL.** Blue channel shows DCadherin. This z-stack begins with the apical confocal sections.
(4.99 MB MOV)

## Abbreviations

AEL: after egg laying
ANK: ankyrin
AP: anterior-posterior
CeN: CD8eGFPNotch
CN: CD8Notch
GFP: green fluorescent protein
Nintra: Notch intracellular domain
