# Supplementary figures and images for: Ligand-Independent Traffic of Notch Buffers Activated Armadillo in Drosophila
Source: PLoS Biol. 2009 Aug 11;7(8):e1000169. doi: 10.1371/journal.pbio.1000169 (PMC2716527; doi:10.1371/journal.pbio.1000169)

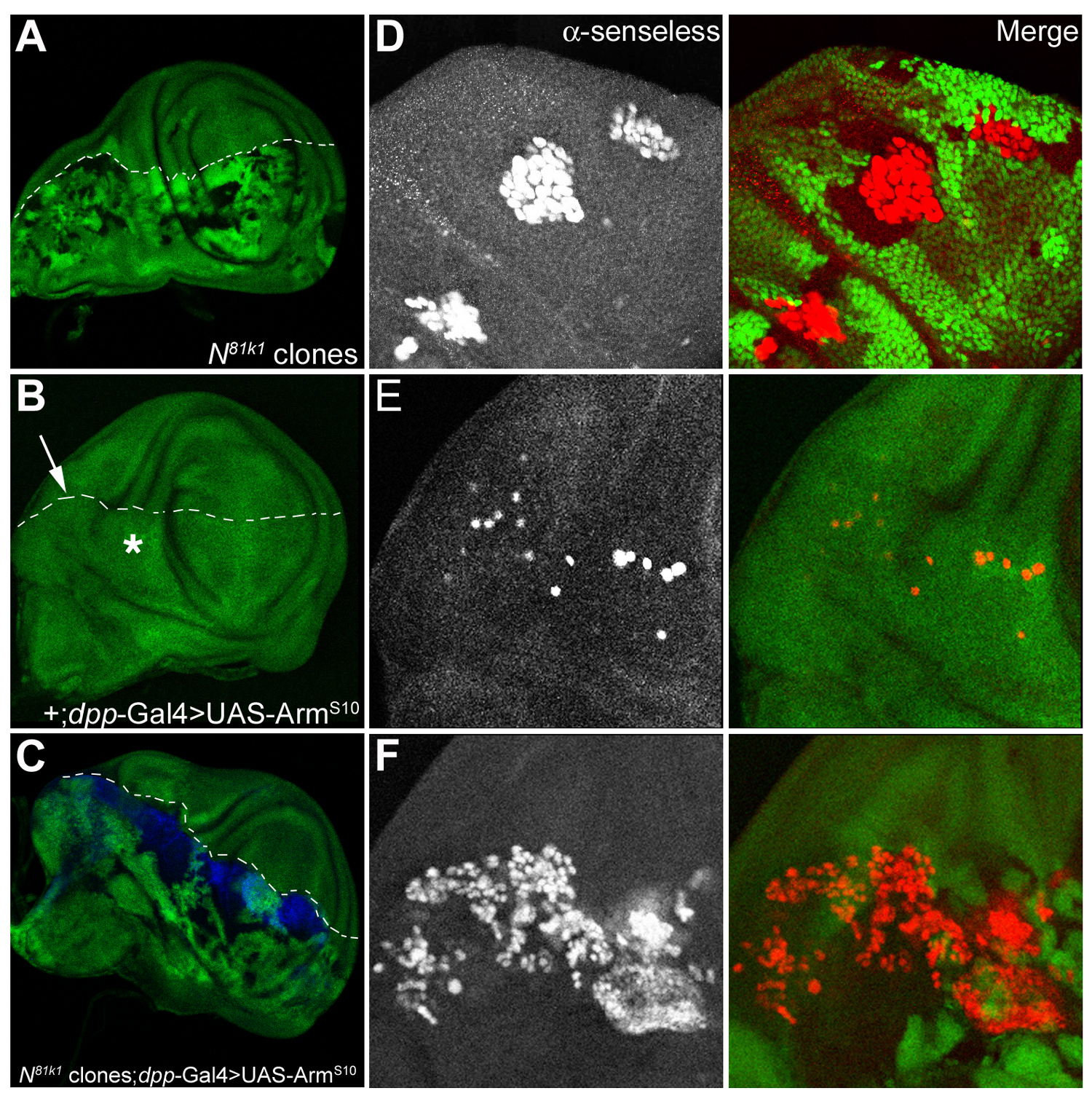

Supplement: Figure S1 — Armadillo induces growth and ectopic neurogenesis in the absence of Notch activity. (A) Wing disc with clones of Notch mutant cells (marked by the absence of GFP, green channel) along the AP boundary generated using the FRT/FLP system. The clones are small, consistent with low growth rate and apoptosis [52],[91]. (B) Wing disc expressing UAS-ArmS10 under the control of dpp-Gal4. Notice the change of morphology that is related to an extension of the hinge (asterisk) and to a small ectopic wing pouch in the scutellar region (white arrow). (C) Wing disc with clones of Notch mutant cells expressing UAS-ArmS10 under the control of dpp-Gal4 (see Materials and Methods for details). Notice that in contrast with (A), the clones are large and also the disc is larger. The comparison of these with the MARCM clones suggests that the ones generated with the FRT/FLP system represent all the stages in the formation of the outgrowths. The images in (A–C) are in the same magnification; the dashed line indicates the AP boundary. The blue channel in (C) shows ArmS10 expression (using α-Myc antibody). (A–C) were taken at the same magnification. (D–F) Higher magnification images of prospective nota showing the expression of Senseless, which labels cells in proneural clusters and represents a high threshold target of Wingless signalling (red channel). (D) In Notch mutant clones, cells within the realm of proneural clusters express Senseless reflecting a failure in lateral inhibition and a high activity of Wingless. (E) Expression of ArmS10 in the notum does not elicit ectopic neural expression. This is in contrast with its effect in the wing pouch where it always elicits ectopic neural expression [43]. (F) In the absence of Notch ArmS10 elicits ectopic expression of Senseless in the notum outside the proneural clusters domain (compare to [D]). (6.84 MB TIF) [file pbio.1000169.s001.tif]

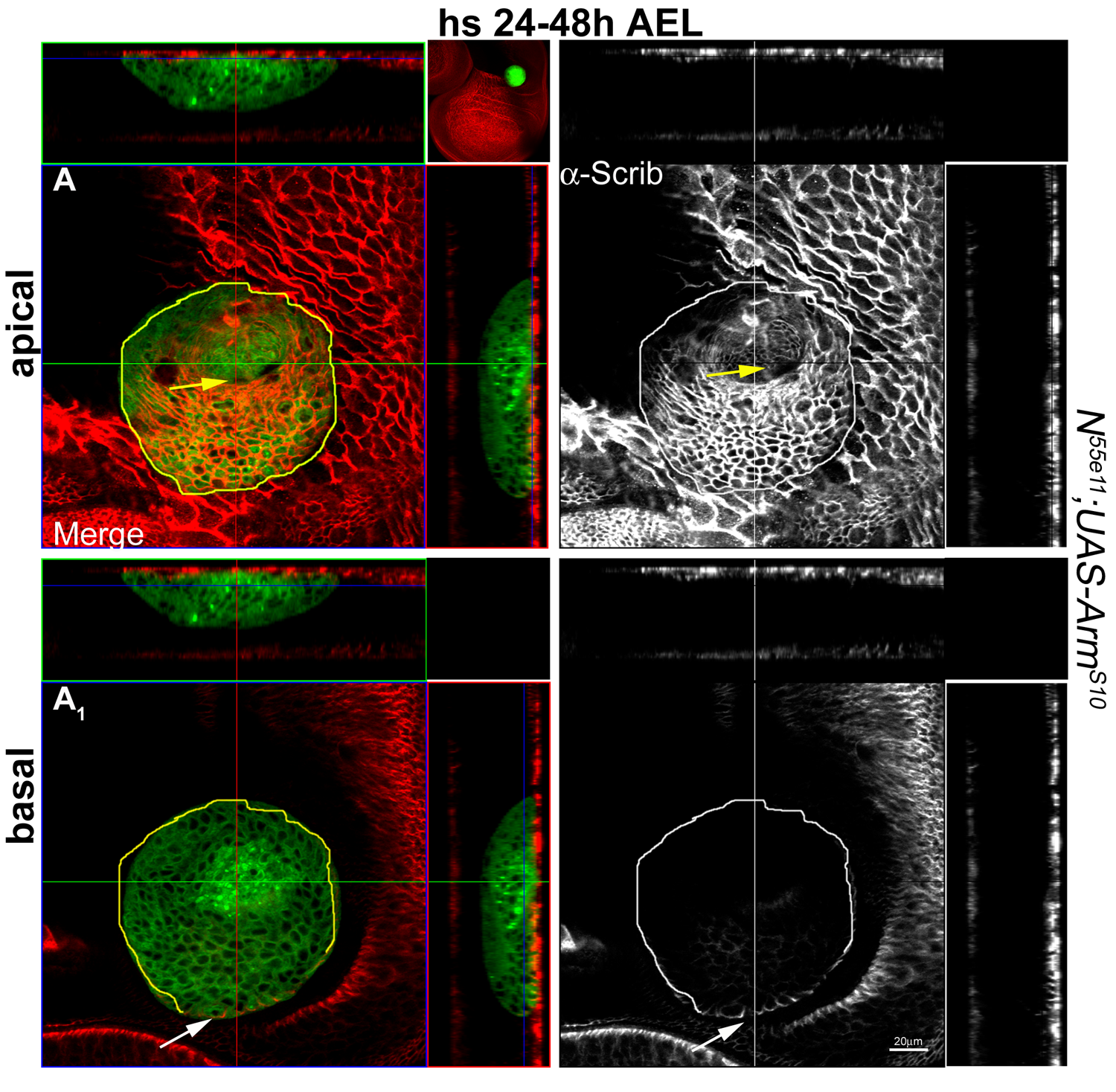

Supplement: Figure S2 — In the absence of Notch, Armadillo induces defects in cell proliferation, adhesion, and polarity at all stages of disc development. Clones induced at 24–48 h of development. Confocal images of third instar wings discs with MARCM clones of Notch mutant cells (labelled in green) that overexpress ArmS10 induced at 24–48 h AEL. (A) is apical, and (A1) is a basolateral section. The red channel shows Scribble (a basolateral cell junction marker). The very dense single sphere of cells is characteristic of these clones and appears to have been engulfed by wild-type cells, which wrap around them. Most of the cells in the sphere have an abnormal polarity as revealed by the loss of Scribble (yellow arrow in [A]). The yellow continuous line marks the position of the clone, in (A1) is interrupted to show clearly the change of polarity of those cells (yellow arrow in [A1]). The complete z-stack of this clone can be found as Video S1. Scale bar, 20 µm. (6.62 MB TIF) [file pbio.1000169.s002.tif]

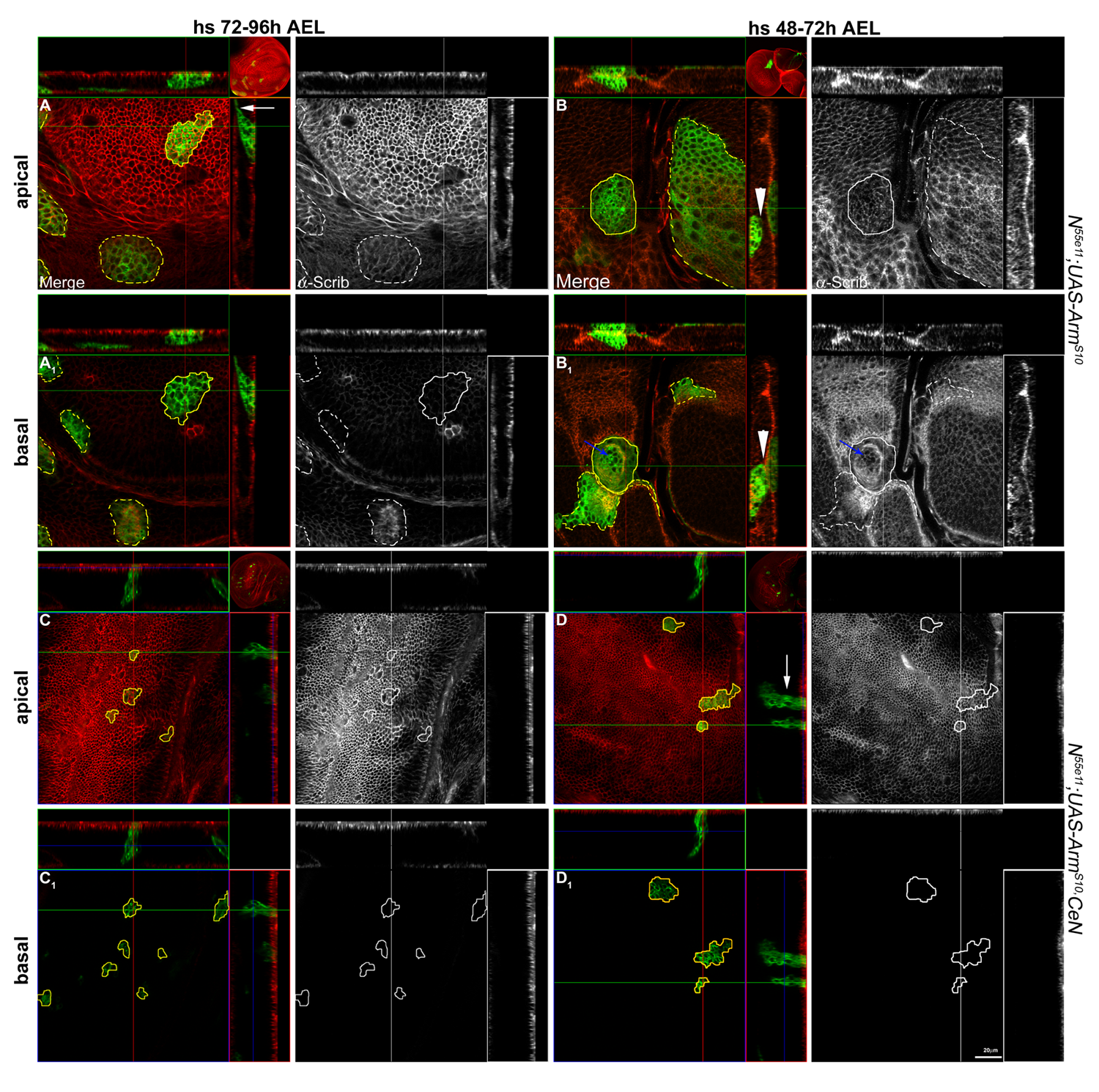

Supplement: Figure S3 — Effect of Notch on the activity of Armadillo in clones of cells of different ages. Confocal images of third instar wings discs with MARCM clones of Notch mutant cells (labelled in green) that overexpress ArmS10 without (A and B) and with (C and D) CeN, induced at 72–96 h (A and C), 48–72 h (B and D). (A–D) are apical, and A1–D1 are basal sections. As in Figure S2, the red channel shows Scribble (a basolateral cell junction marker) and as in other figures, the pictures on the top and the right represent optical z-section through the clones following the green and the red lines shown in the main picture. The small insets at the corners of each image are low magnification pictures of the discs shown, which act as a reference. Note that the size and appearance of the clones change depending on the stage of the induction: the late induced clones are smaller and exhibit an irregular shape (A), while the early ones are bigger and rounder (B). Often the clones lose the basal connexion (white arrow in [A]), some can be seen to coalesce (with one in the peripodial membrane in [B], white arrowheads), and some cells within the clones lose their polarity (blue arrows in [B]). The clones in the wing disc cells are depicted with a continuous line, and those in the peripodial membrane in a dashed line. Expression of CeN rescues the effect of the loss of function of Notch on the activity of ArmS10 (C and D). These clones are smaller, they do not fuse, recover their polarity and span the epithelium. The complete z-stack of (B) and (D) can be found as Videos S2 and S3, respectively. Scale bar, 20 µm. (6.72 MB TIF) [file pbio.1000169.s003.tif]

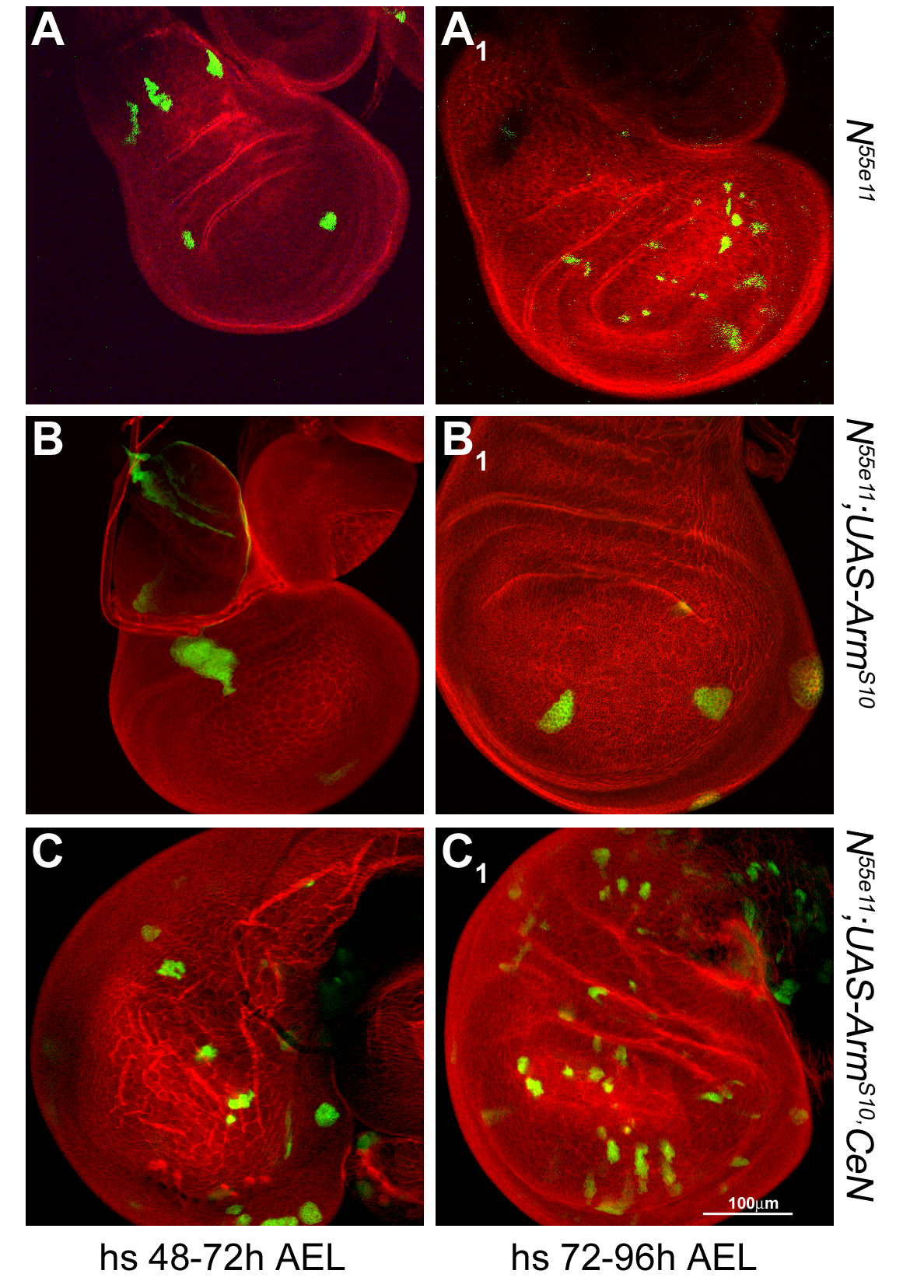

Supplement: Figure S4 — Wing imaginal discs with clones as shown in Figure 2 . Confocal images of third instar wings discs with MARCM clones (labelled in green) of Notch mutant cells (A and A1), of Notch mutant cells that overexpress ArmS10 (B and B1), and of Notch mutant cells that overexpress ArmS10 and CeN (C and C1), induced at 48–72 h (A–C) or 72–96 h AEL (A1–C1). Notice that the clones expressing ArmS10 are much larger and display a rounded appearance. The red channel shows Scribble, the clones are labelled in green. This image corresponds to the discs shown in the inset of Figure 2 and allows a magnified visualization of the distribution and shape of the clones in the imaginal discs. Scale bar in (C1), 100 µm. (5.91 MB TIF) [file pbio.1000169.s004.tif]

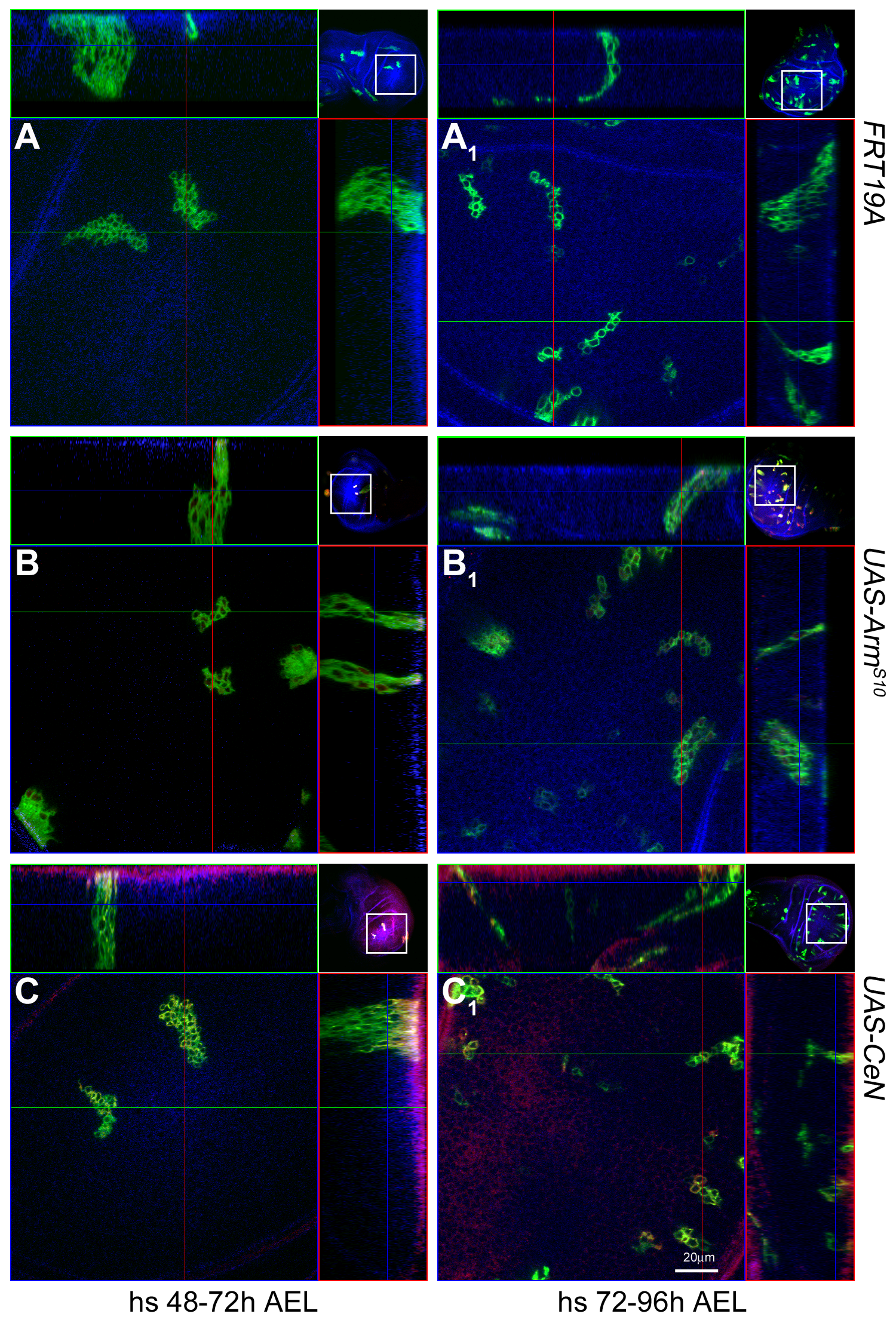

Supplement: Figure S5 — MARCM clones of cells with different genotypes. Confocal images of third instar wings discs with MARCM clones of wild-type cells (induced in the FRT19 background, see Materials and Methods for details) (A and A1), ArmS10 (B and B1), or CeN (C and C1) (labelled in green) induced at 48–72 h AEL (A–C) 72–96 h AEL (A1–C1). The wild-type clones exhibit the well-described appearance with an elongation across the dorsal-ventral (DV) axis. Clones of cells expression ArmS10 do not exhibit increased growth, though they appear smaller and a bit more rounded. They still maintain the apico-basal polarity (compare with the effects of expressing ArmS10 in the absence of Notch, e.g., Figures 2 and S7). The Myc label (red) highlights the nuclear localization of ArmS10 under these conditions. The clones of cells expressing CeN are smaller than wild type and show a tendency to be more rounded. The blue channel shows the DCadherin staining; the red is α-Myc in B and B1, and α-NICD in C and C1. Scale bar, 20 µm. (10.00 MB TIF) [file pbio.1000169.s005.tif]

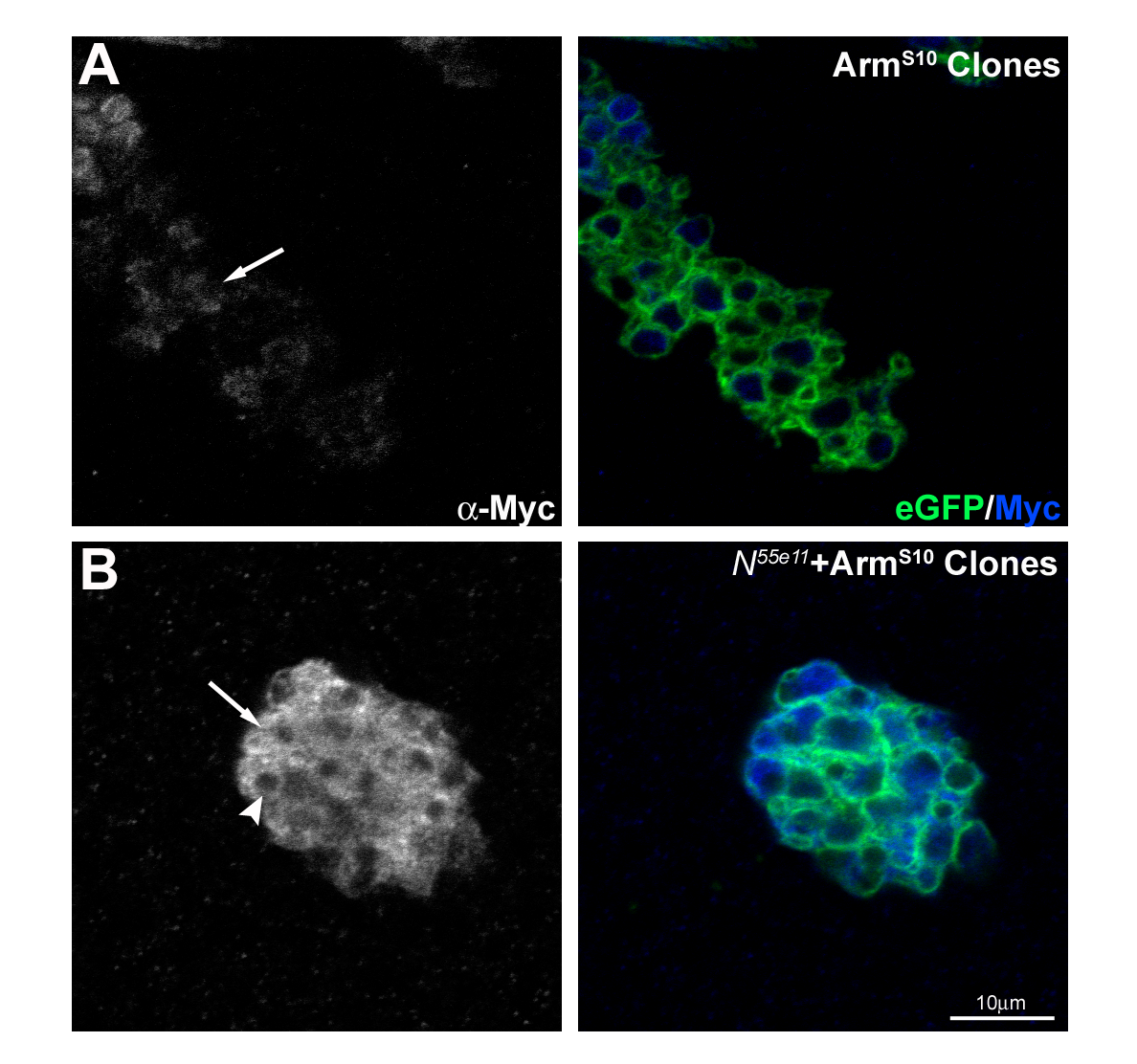

Supplement: Figure S6 — Activated Armadillo is stabilized in the absence of Notch. Confocal images of third instar wings discs expressing ArmS10 in clones in the presence (A) and absence (B) of Notch. (A) Wing disc with a clone of cells that overexpress ArmS10 (labelled in green) induced at 72–96 h AEL. (B) Wing disc with a MARCM clone of Notch mutant cells that overexpress ArmS10 (labelled in green) induced at 72–96 h AEL. The blue channel shows ArmS10 expression (using α-Myc antibody), the green highlights the membrane and the cortex. Both images were taken under the same confocal conditions at the level of the nuclei. Note that in the absence of Notch there are increased levels of ArmS10, which now can be observed prominently in the nuclei (arrows). A small region of the nucleus is devoid of staining in some sections (arrowhead); this region probably corresponds to the nucleolus. Scale bar, 10 µm. (3.97 MB TIF) [file pbio.1000169.s006.tif]

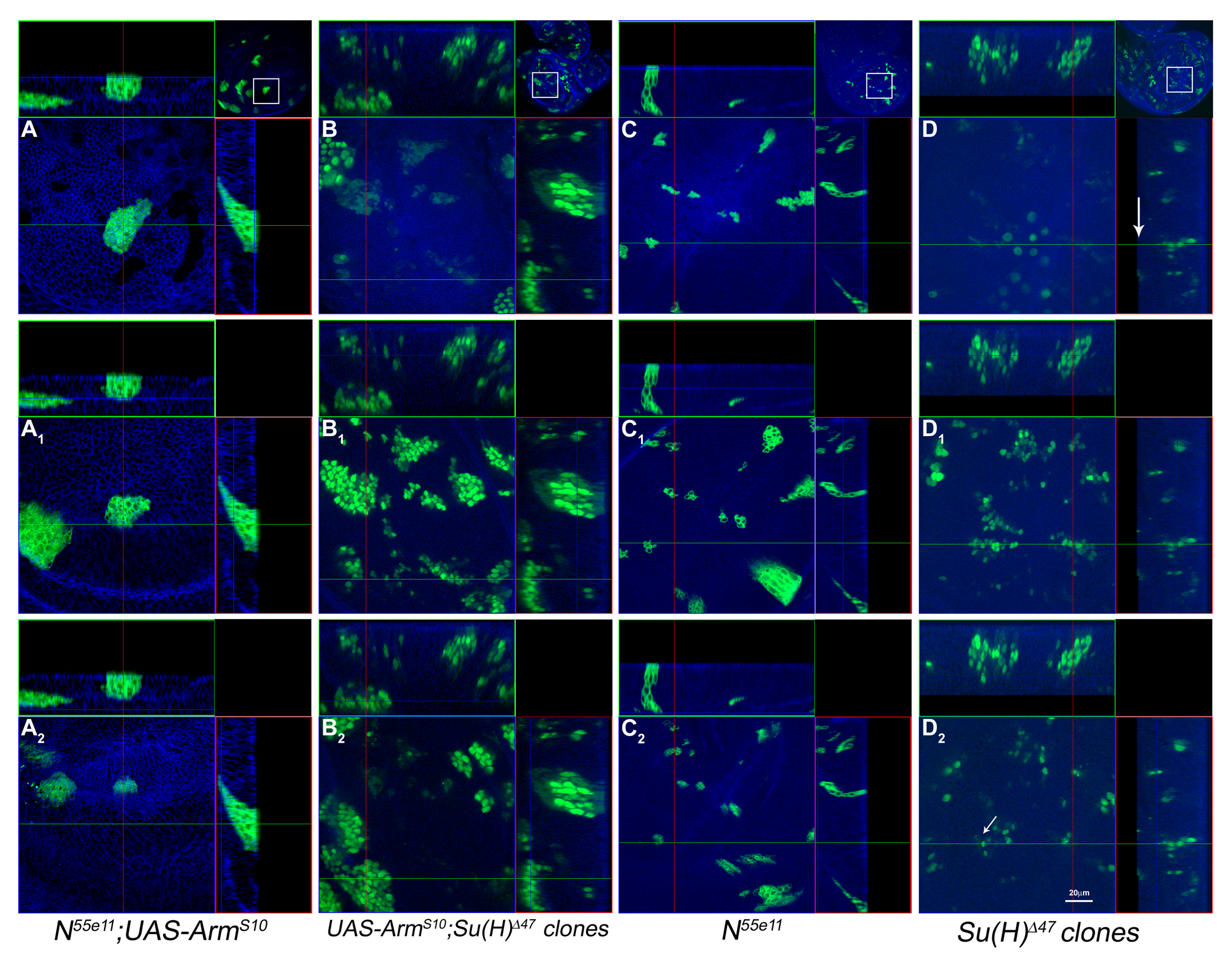

Supplement: Figure S7 — Comparison of the Notch and Su(H) mutant clones with and without expression of ArmS10. Confocal images of third instar wings discs with clones of cells induced between 72–96 h AEL mutant for both Notch55e11 (A and C) or Su(H) Δ47 (B and D), without and with ArmS10 as indicated (labelled in green). This late generated Su(H) Δ47 clones of cells already have apoptotic cells on the basal side (white arrows), which are not seen in the Notch55e11. Expression of ArmS10 in these cells increases the size of the Su(H) mutant clones, alters their appearance, and reduces, but does not eliminate, the number of apoptotic cells in the basal region or the interdispersion of the clones. In the Notch mutant clones, the expression of ArmS10 increases the size of them and there are fewer (probably due to the coalescence of them) Technical details of the images as in Figure 2 with the small insets at the corners of each image are low magnification pictures of the discs shown which act as a reference. The complete z-stack of (B) and (D) can be found as Videos S4 and S5. Scale bar in (D2), 20 µm. (9.62 MB TIF) [file pbio.1000169.s007.tif]

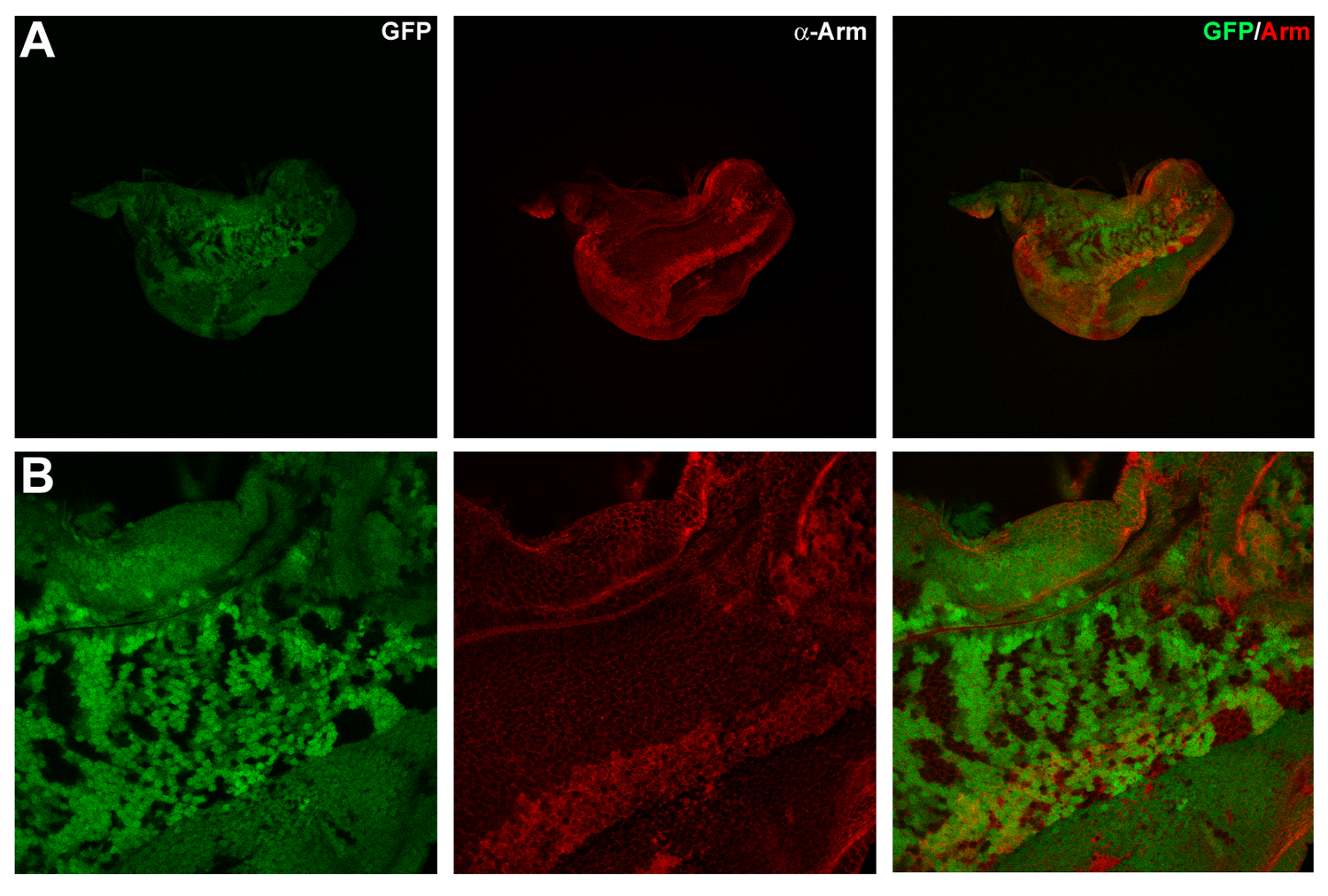

Supplement: Figure S8 — Effects of Nintra on the activity of ArmS10 in the absence of Notch. Third larval instar imaginal disc expressing UAS-ArmS10 and Nintra under the control of dpp-Gal4, and with clones of cells mutant for N81k1 generated using the FRT/FLP in the same manner as those in Figures 1 and 5 (and see Materials and Methods for details). (A) Image taken with a 10× objective; (B) higher magnification showing details of the clones. A comparison with Figure 5 shows that Nintra rescues the size of the clones. The discs are very large and elongated in the dorsal-ventral (DV) direction due to the effects of the interaction between Nintra and ArmS10 in the induction of the primordium [53]. It is worth pointing out that this interaction is amplified in this genetic background in which there is only one dose of Notch. It is also important to mention that the well-established interaction between Nintra and Arm cannot explain the suppression of the activity of Arm, which we observe in the clones. We surmise that this inhibition is mediated by squelching of Arm by the excess Nintra (see text for further details). (4.56 MB TIF) [file pbio.1000169.s008.tif]

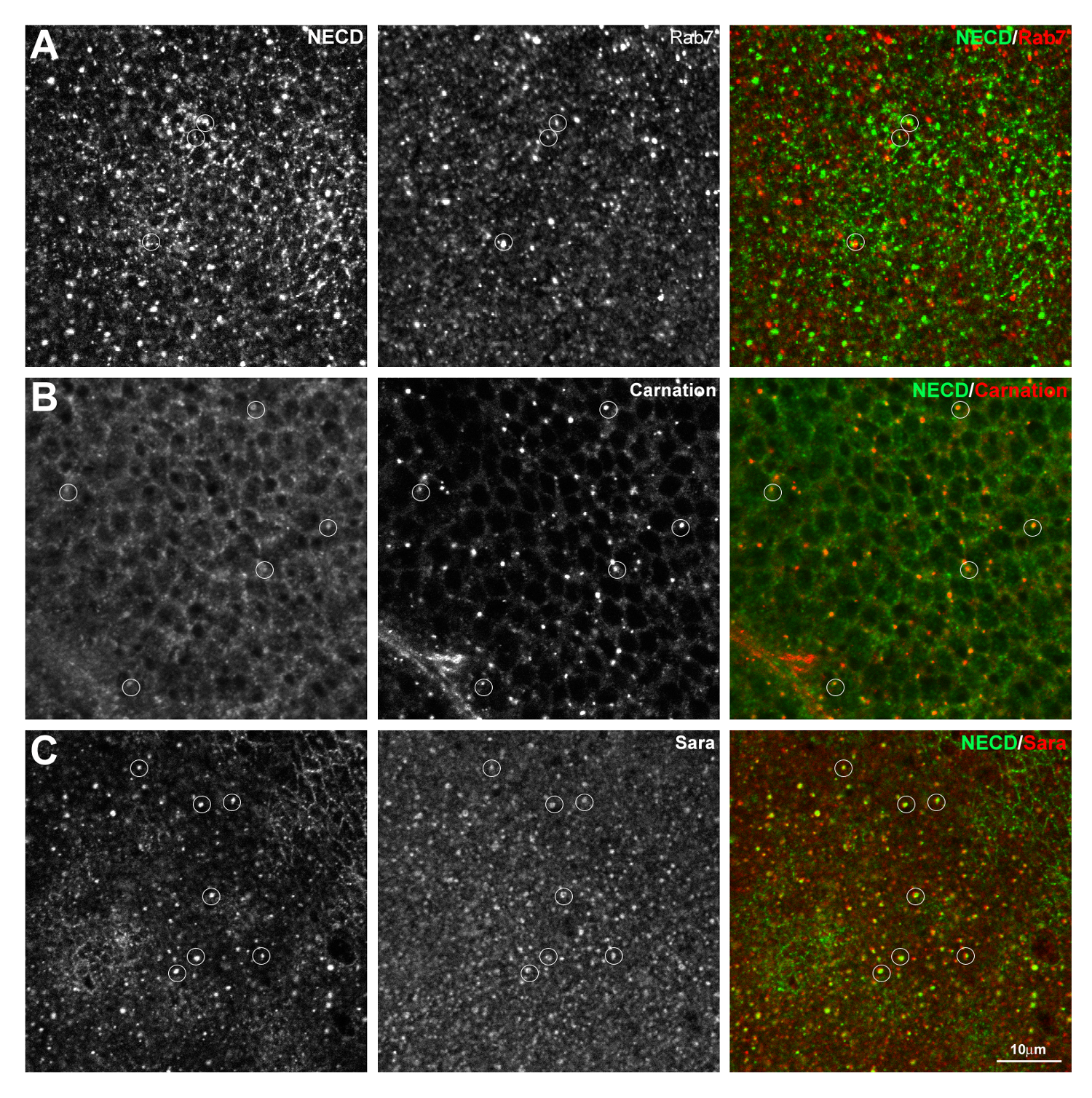

Supplement: Figure S9 — Notch colocalizes with some endosomal markers. Image of third instar wing discs fixed and stained for NECD (green channel) and Rab7 (A), Carnation (B), or Sara (C) (red channel). (A) and (C) are subapical sections and (B) is 7 µm underneath the level of the adherens junctions. Note that there are some vesicles in which NECD and Rab7 colocalize, more with Carnation and even more (nearly all) with Sara. Scale bar, 10 µm. Circles highlight some of the vesicles with colocalized stain. (6.80 MB TIF) [file pbio.1000169.s009.tif]

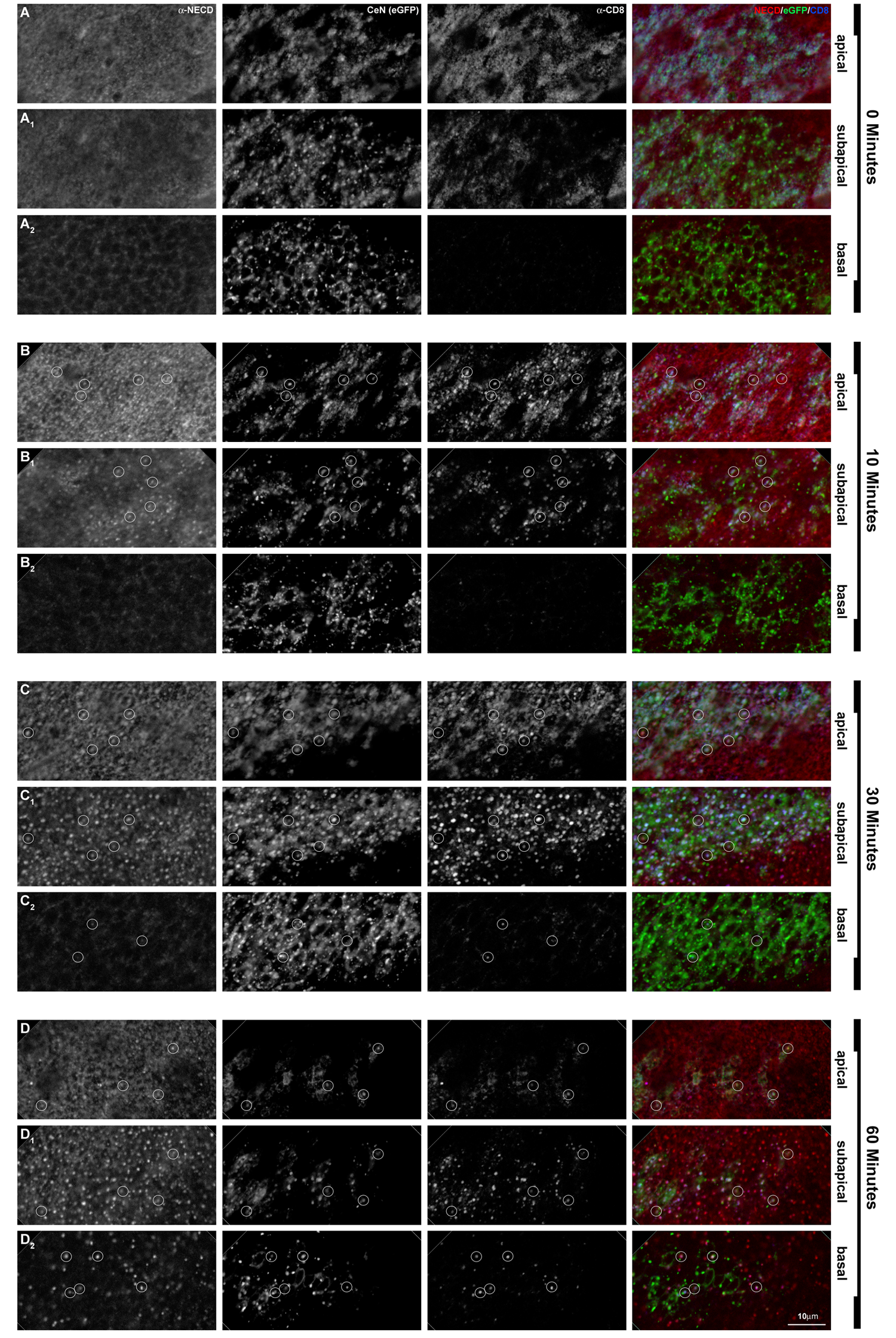

Supplement: Figure S10 — Endocytosis and traffic of CeN and Notch (extended version of Figure 6 ). (A–D) Notch and CD8 tracked over time after pulsing live wing discs expressing CeN with dpp-Gal4 with an antibody against the extracellular domain of Notch (red channel) and CD8 (blue channel), and chasing for 0 (A), 10 (B), 30 (C), and 60 min (D) (details in Materials and Methods). (A–D) are apical sections at the level of the adherens junctions level; (B1–D1) are subapical sections, 1 µm underneath; and (B2–D2) are basal sections, 7 µm underneath the apical ones. After 0 min of chasing, most of the labelled endogenous Notch and the expressed CD8 localize to the apical membrane of the cells (A) and there are no vesicles in subapical or basal levels (A–A2). After 10 min of chase, both Notch and CeN localize in apical and subapical dots that correspond to vesicles (B–B2). After 30 min, the endogenous Notch and CeN have been cleared almost completely from the apical membranes and can be found mostly in subapical vesicles and also now in the basal domain (C–C2). After 60 min of chase, there is no cell surface labelled and the endogenous Notch localizes in vesicles in apical, subapical, and basal levels; at this time point, CD8 also goes to apical vesicles, but mainly in the subapical and basal levels (D–D2). The overall levels have decreased. In all cases the apical and basal images were taken in equivalent levels in the dorsal region of the wing pouch. The GFP highlights the steady state CeN against the background of the dynamic experiment. Scale bar, 10 µm. Circles highlight colocalized stain. (4.30 MB TIF) [file pbio.1000169.s010.tif]

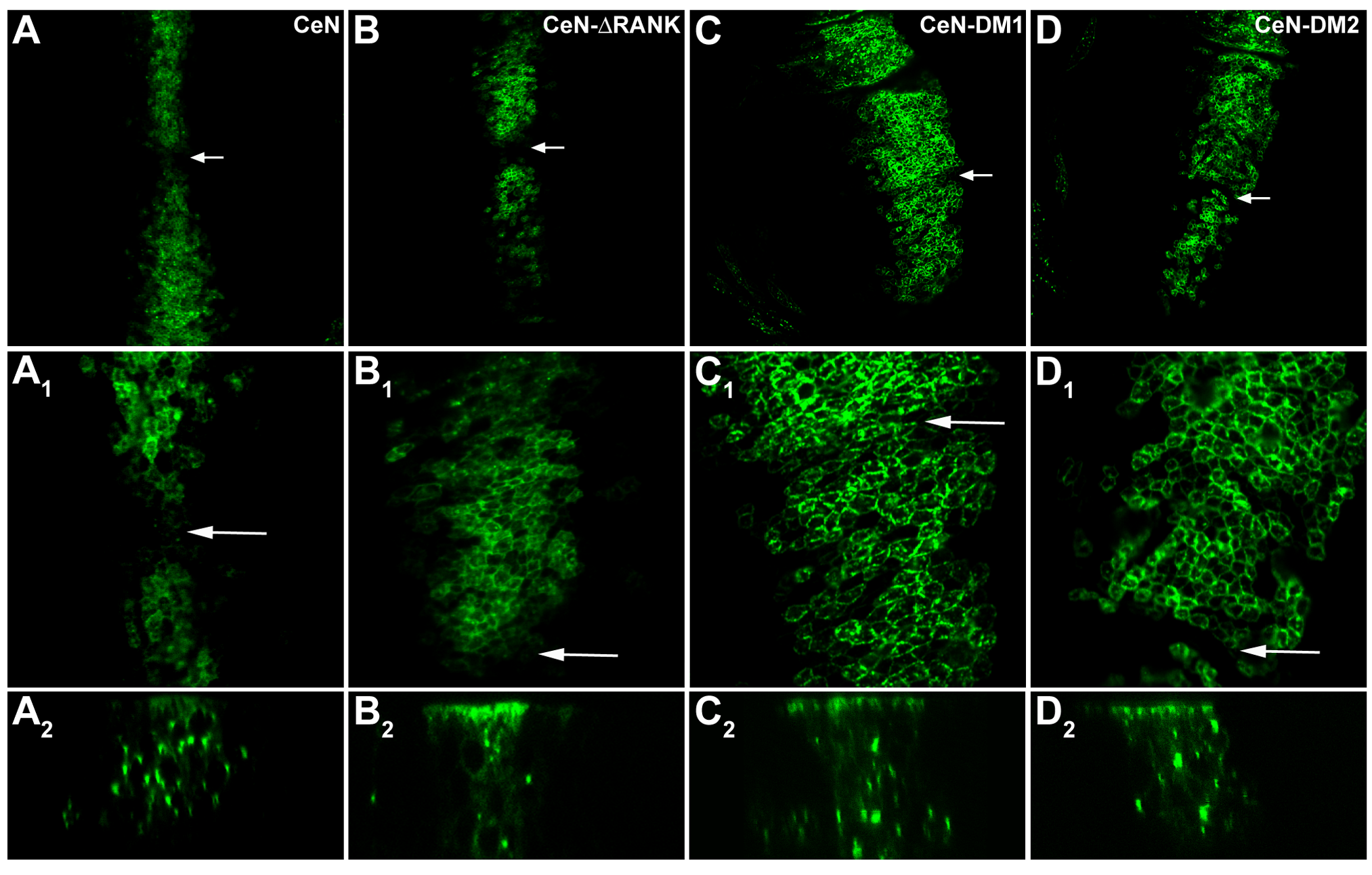

Supplement: Figure S11 — Mutations in the ANK domain impair Notch traffic. Wing pouch images from third instar discs expressing CeN (A–A2), CeN-ΔRANK (B–B2), CeN-DM1. (C–C2) and CeN-DM2 (D–D2) under the control of dpp-Gal4. All are derivatives of CeN; CeN-ΔRANK is a deletion of the RAM and ANK domains of the intracellular domain, whereas CeN-DM1 and CeN-DM2 are point mutations in the fourth and fifth ANK repeats (for details see Materials and Methods). All images were taken from the apical level of the wing pouch; the arrows point to the dorsal-ventral (DV) boundary; the eGFP fluorescence (from the CeN molecule) is shown in all cases. (A2–D2) are confocal optical z-sections through the wing pouches. Note that there is an apical accumulation of the CeN mutant molecules in apical levels, particularly clear in the CeN-ΔRANK and fewer vesicles in basal levels, which tend to be of larger size. These are images from a steady state, for pulse chase of one example see Figure S12. (7.86 MB TIF) [file pbio.1000169.s011.tif]

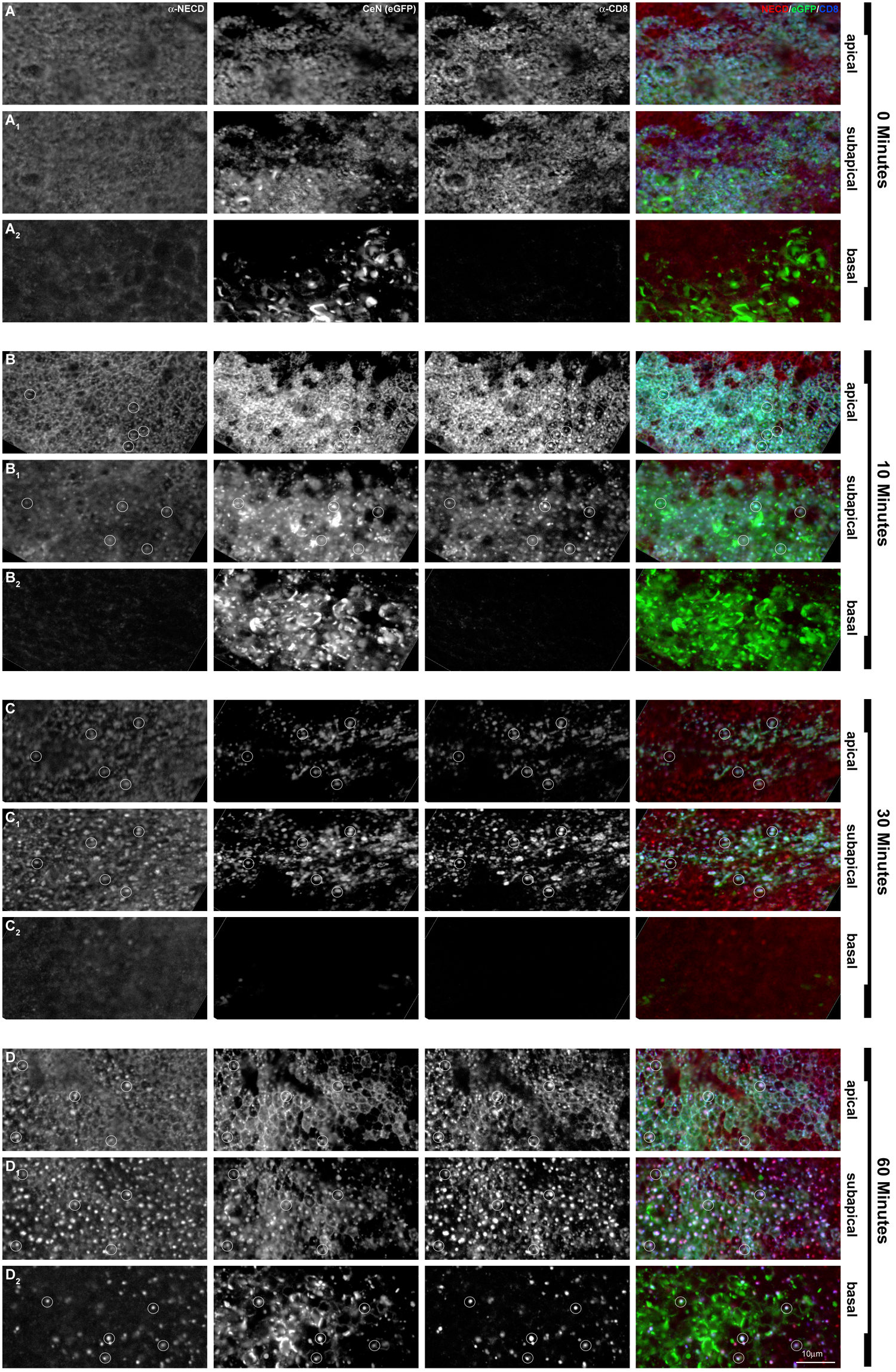

Supplement: Figure S12 — Endocytosis and traffic of CeN-DM1. CeN-DM1 (see Figure S10) exhibits a Notch-related distribution and although it is endocytosed, its dynamics are much slower than CeN and than Notch, as revealed by the observation that in antibody uptake experiments it remains at the cell surface for longer than CeN. (A–D) Notch and CD8 (from CeN-DM1) tracked over time in the same cells by pulsing CeN-DM1 expressing live wing discs with an antibody against the extracellular domain of Notch (red channel) and CD8 (blue channel), and chasing for 0 (A), 10 (B), 30 (C), and 60 min (D). (A–D, apical; B1–D1, subapical sections; B2–D2, basal sections; these sections were taken at the same levels as the ones in Figure S10). After 0 min of chasing, the endogenous Notch and CD8 localize in the apical membrane of the cells (A) and there are no vesicles in subapical or basal levels (A–A2). After 10 min of chase, Notch and CeNDM1 can mainly still be found in the apical level (suggesting that it remains there for longer time), and some in subapical vesicles (B–B2). After 30 min, the endogenous Notch and CeNDM1 have been cleared almost completely from the apical membranes and can be found mostly in subapical vesicles that look bigger than the CeN ones. By this time the wild-type Notch and CeN (see Figure S10) can be detected in the basal domain but not CeN-DM1 (C–C2). After 60 min of chase, the endogenous Notch and CeN-DM1 localizes in apical, subapical, and basal levels; at this time point, CD8 also goes into vesicles, but mainly in the apical and subapical levels (D–D2). In all cases the apical and basal images were taken in equivalent levels in the dorsal region of the wing pouch. Notice the large amount of CeN-DM1 that is present at this stage, which contrasts with the lower levels of CeN (see Figure S10). Scale bar, 10 µm. Circles highlight colocalized stain. All the images of Figures 6, S10, and S11 were taken under the same confocal conditions and processed equivalently, so that we can com [file pbio.1000169.s012.tif]

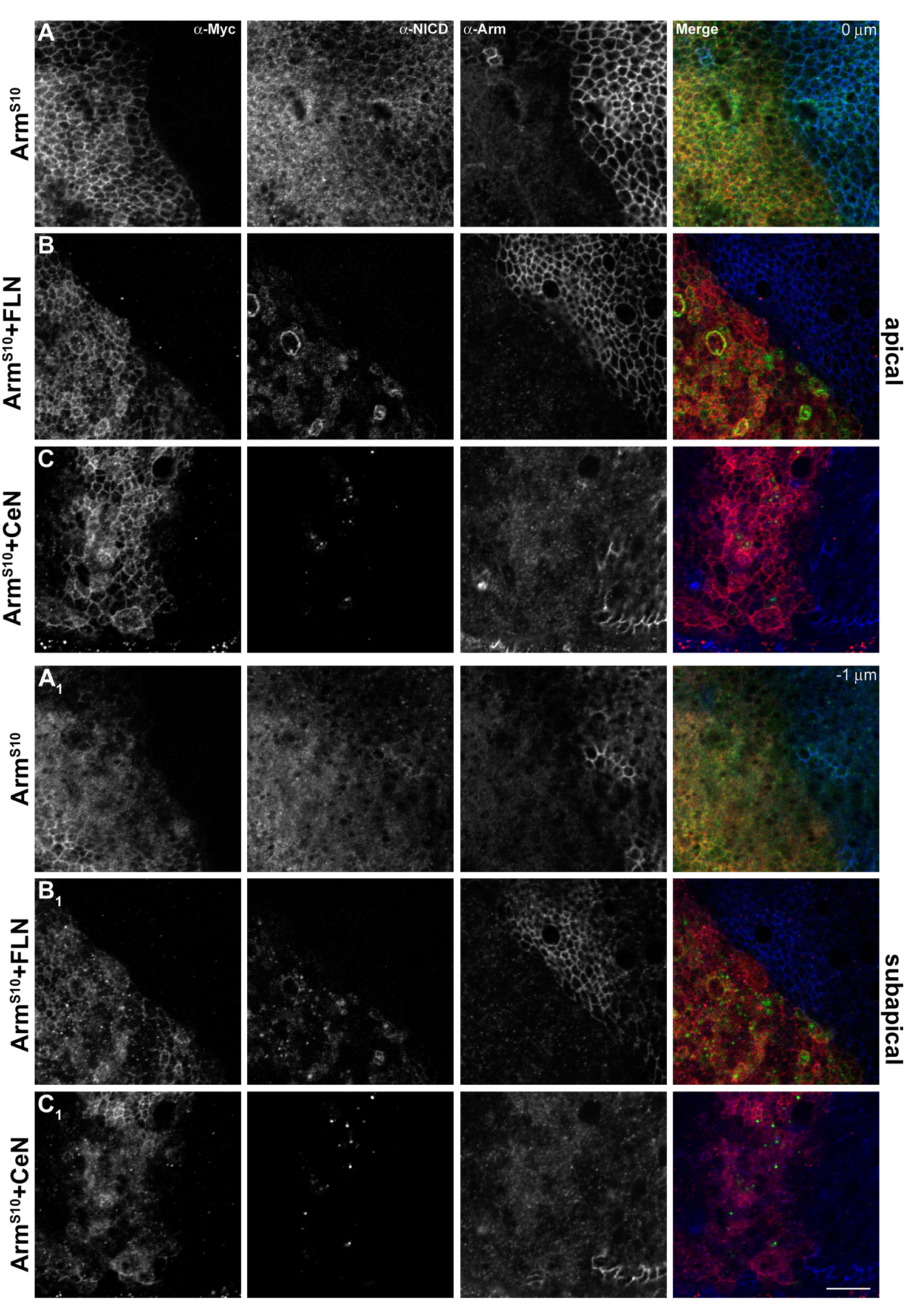

Supplement: Figure S13 — Armadillo and Notch induce reciprocal alterations in their subcellular localization. Analysis of the distribution and localization of ArmS10 (red channel), NICD (in green), and endogenous Arm (blue) in wing discs expressing UAS-ArmS10 (A); UAS-ArmS10;UAS-FLN (B); UAS-ArmS10,UAS-CeN (C), under the control of dpp-Gal4. Apical (A–C) and subapical (A1–C1; 1 µm below the apical section) confocal sections of the dorsal region of the wing pouch are shown. The expression of ArmS10 promotes an accumulation of endogenous Notch apically and subapically as revealed by increased diffused staining with anti-NICD antibody (A). This accumulation is probably due to the stability of ArmS10, which tends to reside in the apical region, apparently have a slower turn over, and thereby stabilize Notch in that region. This result can be seen apically and is particularly obvious subapically, where a large accumulation of Notch can be observed. Expression of both FLN and CeN with ArmS10 lead to changes in the distribution and appearance of both ArmS10 and endogenous Arm. Most significantly, overexpressed Notch induces a decrease in the amount of ArmS10 in the apical surface and its accumulation in the subapical in a diffuse form with some vesicles (compare [A] with [B] and [C]). This effect can also be seen in the endogenous Arm: ArmS10 displaces it from the apical membrane towards a subapical “shadow” that is gathered into vesicles by FLN and CeN both in apical and subapical sections (B–B1 and C–C2). Scale bar, 10 µm. (9.77 MB TIF) [file pbio.1000169.s013.tif]
